# The Rose Model of Water: Linking Theory and Simulation

**DOI:** 10.3390/e28060682

**Published:** 2026-06-12

**Authors:** Peter Ogrin, Tomaz Urbic

**Affiliations:** Faculty of Chemistry and Chemical Technology, University of Ljubljana, Vecna Pot 113, SI-1000 Ljubljana, Slovenia

**Keywords:** water model, thermodynamics, phase diagram

## Abstract

Water plays a fundamental role in countless natural and technological systems, where its unique properties are connected with those of the surrounding environment. The water’s anomalous behaviors arise from the directional nature of hydrogen bonding between molecules. To understand these anomalies, numerous molecular models have been developed, ranging from detailed atomistic descriptions to coarse-grained, conceptually simple representations. Among the latter, the two-dimensional Rose model offers a minimal yet physically meaningful framework that reproduces key thermodynamic and structural anomalies of real water while remaining analytically tractable. In this work, we present a comprehensive review and comparison of results obtained for the Rose water model using Monte Carlo and molecular dynamics simulations, thermodynamic perturbation theory, integral equation theory (both orientation-averaged and orientation-dependent), and an analytical model. The study encompasses the thermodynamic and structural properties of pure Rose water and of systems containing nonpolar solutes. Moreover, the anomalous regions and phase behavior of the model are systematically explored. The combined results demonstrate that the Rose model successfully captures the essential physics of water’s anomalies within a simple and computationally efficient framework, providing a valuable bridge between theory and simulation.

## 1. Introduction

Water is likely the most extensively researched substance and has been the subject of investigation for centuries, as its fundamental role in both biological and non-biological systems has long been recognized. Water’s special importance arises from the combination of its abundance and a variety of anomalous properties [1], which strongly influence nearly every system in which it is present. The anomalous behavior of water is evident in its thermodynamic properties (such as the density maximum at 4 °C, the negative thermal expansion coefficient, and the unusually large heat capacity of the liquid), dynamic properties (such as the non-monotonic temperature dependence of the diffusion coefficient [2,3,4,5,6,7]), and structural properties (including the remarkably rich phase diagram with 20 known crystalline ice forms [8,9,10], and regions of unusual local structural order [11]).

Although water exhibits a wide range of anomalous properties, their underlying origin is generally attributed to the ability of water molecules to form hydrogen bonds. The locally tetrahedral arrangement of water molecules is believed to be closely connected with many of these anomalies [12,13]. Evidence for this link is provided by the fact that other liquids with tetrahedral local structures also display similar anomalous behavior [14,15]. Examples include molten $GeO_{2}$ [16], liquid $BeF_{2}$ [17,18], liquid silicon [19], and liquid silica [20,21]. This indicates that the local structural ordering of water molecules plays a crucial role in nearly all of its (anomalous) properties.

The properties of water confined at the nanoscale deviate from those of bulk water. For example, in monolayer confinement, the dielectric constant of water decreases [22], the phase behavior differs considerably from that of bulk water [23,24,25,26,27,28,29], the freezing point is altered [30,31], and diffusion is enhanced [32]; while confined water retains anomalous thermodynamic behavior comparable to that of the bulk, these anomalous regions shift to lower temperatures, and its structural and dynamical features resemble those of bulk water at higher temperatures [33]. Moreover, ab initio studies suggest that water monolayers can adopt structural motifs belonging to either a square or honeycomb hydrogen-bonded network [34], and a liquid–liquid phase transition has also been predicted at low temperatures [35]. In short, confinement modifies the properties of water monolayers quantitatively, for example, by shifting phase transitions and anomalous regions, but the qualitative behavior remains consistent with bulk water, as it still forms the same hydrogen-bonding interactions that make water such a unique liquid.

The two-dimensional model presented in this work could, in principle, also be employed to represent monolayers. Nevertheless, the particular model used here has not been designed or validated for the description of water monolayers. A crucial distinction is that monolayers are subject to nanoscale confinement, which limits molecular motion and reduces available degrees of freedom. In contrast, a two-dimensional water model is not inherently confined within 2D space and thus cannot be directly interpreted as representing a water monolayer. Rather, such models are generally regarded as simplified 2D analogues of water that reproduce certain essential properties of real, three-dimensional liquid water.

Many different water models have been developed in attempts to capture the unique behavior of water in various systems. The Bernal–Fowler model [36] was the first realistic water model, using point charges and a repulsion term. As computational sciences developed, similar models began to be used in simulations [37]. The models from the TIP [38,39,40,41,42,43] and SPC [44,45] families were developed some time ago for use in simulations of water and aqueous systems and are still widely used today. Most of these models are empirical potentials parametrized to replicate the properties of experimental water. These models consist of a Lennard–Jones potential and differently placed point charges; the interactions of these point charges are also responsible for hydrogen bonding. More recently, more complex and accurate models have been developed, enabling accurate calculation of a wide range of properties and effects that were neglected in classical empirical models. Such advanced models often rely on ab initio quantum mechanical calculations for high accuracy and machine learning methods to speed up the calculations [46,47,48,49,50,51].

The crucial properties of water can also be captured with much simpler models that lack atomistic detail. The main strength of simple models lies in their ability to investigate the fundamental physical origins and mechanisms underlying diverse phenomena. By stripping away secondary effects and interdependencies, such models allow the relationships between the studied properties to emerge more transparently. Their simplicity also makes them valuable tools for developing and testing new computational methods of varying complexity, from advanced machine learning algorithms to statistical mechanical theories at different levels of sophistication. In addition, when the model is formulated in two dimensions, the resulting structures are easier to visualize, enabling clearer identification of structural changes and effects.

Well-known examples of such two-dimensional models include the Mercedes–Benz (MB) model [52,53] and the Rose water model [54]. These two models are closely related, as the Rose model was introduced as a computationally more efficient mimic of the MB model. Another representative of this class is the coarse-grained mW water model [55], which, like the MB model, neglects long-range electrostatic interactions and instead incorporates hydrogen bonding by explicitly favoring tetrahedral molecular arrangements. More recently, another coarse-grained model has been proposed, also based on explicit tetrahedral hydrogen bonding [56].

In this article, we review the research conducted using the Rose water model. The Rose water model is a simple water-like model that describes water molecules as two-dimensional Lennard–Jones disks. The disks have an explicit hydrogen bonding potential added, enabling the formation of hydrogen bonds between particles. The hydrogen-bonding potential has an orientation-dependent term that uses the Rose function, from which the model gets its name. Due to the formulation of the model, each interacting molecule has an independent contribution to the hydrogen bond energy. Consequently, hydrogen half-bonds can be formed, which are not physically correct. Two different parametrizations of the Rose model have been most commonly used. One parametrization, called the MB parametrization, is such that the properties of the model are more similar to the MB model, while the other parametrization, called the real parametrization, more closely resembles real experimental water.

The Rose model qualitatively predicts the anomalous properties of pure water and the anomalous solvation thermodynamics of nonpolar solutes [57]. Wertheim’s thermodynamic perturbation theory (TPT) and integral equation theory (IET) were applied to the Rose water model. The IET successfully predicted pair correlation functions between Rose particles, while thermodynamic properties calculated with IET and TPT were in semi-quantitative agreement with those obtained from Monte Carlo (MC) simulations [57]. To improve IET results, an orientation-dependent IET was developed for the Rose water model [58]. Incorporating orientational dependence in IET improves agreement between theoretical and simulation results and enables the calculation of orientation-dependent properties such as spatial distribution functions. The orientation-dependent IET was also extended to include nonpolar solutes in water [59]. Properties of systems with nonpolar solutes of different sizes solvated in Rose particles were calculated by the theory and compared to simulations; the effect of nonpolar solutes on the local structure of the solution was also investigated [59]. The system of nonpolar solutes in water modeled with the Rose water model was also studied using TPT, focusing on thermodynamic properties [60]. Using MC simulations, the properties of the Rose model were investigated over a wide range of conditions, and regions of anomalous properties were identified. Based on the size of these regions, the anomalous properties were ordered into a hierarchy [61]. The phase behavior of the Rose water model was also investigated. Using MC simulations and TPT, the liquid part of the phase diagram was determined [62]. In search of an automatic method for easy determination of the phase diagram from MD simulation data, the entire phase diagram of the Rose model was also determined. First, the phase diagram was determined based on data obtained by nested sampling (NS) and molecular dynamics [63,64]. Subsequently, an unsupervised machine learning approach was developed to determine the phase diagram of the Rose model in a more automated way [64]. The behavior of the Rose water model confined in random porous media was investigated using associative replica Ornstein–Zernike theory [65], and the results were compared to MC simulations. The effects of static and alternating electric fields on the properties of the Rose water model were also studied using MD simulations [66,67]. When the model was exposed to an alternating electric field, an interesting resonance effect was observed. To describe this effect, a model connecting hydrogen bonding and resonance in an electric field was developed [67].

Conceptually based on the Rose model and MB model, an analytical model of water was developed. This model enables the calculation of thermodynamic, dynamic, and structural properties of water [68]. The model successfully predicts all the properties obtained from simulations of the Rose model, but with only a fraction of the computational time.

In this article, we review the calculations and results for the Rose water model, focusing on the real parametrization of the model. The same properties of the Rose model calculated using different methods are collected and compared to highlight the advantages and disadvantages of each method. This paper is organized as follows. In the next section, the Rose model is described (Section 2). In Section 3, the different methods used to calculate the properties of the Rose model are outlined. The results are presented and discussed in Section 4 and summarized in Section 5.

## 2. The Model

The Rose water model is a simple two-dimensional water model. Its name originates from the use of Rose functions in the hydrogen bonding potential [54]. In this model, each water molecule is represented as a two-dimensional Lennard–Jones (LJ) disk augmented with a hydrogen bonding potential that enables directional bonding:

(1) $$U{\left({\vec{X}}_{i},{\vec{X}}_{j}\right)}_{\mathrm{rose}}=U_{LJ}\left(r_{ij}\right)+U_{HB}\left({\vec{X}}_{i},{\vec{X}}_{j}\right),$$

where $r_{ij}$ is the distance between the centers of molecules *i* and *j*, and ${\vec{X}}_{i}$ and ${\vec{X}}_{j}$ are vectors describing the positions and orientations of molecules *i* and *j*. The LJ interaction has its standard form:

(2) $$U_{LJ}\left(r_{ij}\right)=4\epsilon_{LJ}\left[{\left(\frac{\sigma_{LJ}}{r_{ij}}\right)}^{12}-{\left(\frac{\sigma_{LJ}}{r_{ij}}\right)}^{6}\right].$$

Each molecule contributes independently to the hydrogen bonding potential. Specifically, the contribution from molecule *i* is independent of molecule *j*, and vice versa:

(3) $$U_{HB}\left({\vec{X}}_{i},{\vec{X}}_{j}\right)=U_{HB}\left({\vec{r}}_{ij}\right)+U_{HB}\left({\vec{r}}_{ji}\right).$$

Because each molecule contributes independently to the hydrogen-bonding potential, the model formally allows the formation of half hydrogen bonds, where one molecule is oriented favorably for bonding while the partner is not. These configurations are unphysical in real water, where hydrogen bonds are inherently cooperative. However, their energetic contribution is limited because the total hydrogen-bond energy is halved compared to a fully formed bond, and their statistical weight is small at typical simulation temperatures. As a result, half bonds slightly broaden the angular and distance distributions of hydrogen-bonded pairs but do not significantly alter the overall structural or thermodynamic behavior of the liquid. This simplification is retained to maintain computational efficiency and analytical tractability of the model. The hydrogen bonding contribution from a single molecule is the product of a distance-dependent term $s(r_{ij})$ and an orientation-dependent term $U(\theta_{ij})$:

(4) $$U_{HB}\left({\vec{r}}_{ij}\right)=\frac{\epsilon_{HB}}{2}\;s\left(r_{ij}\right)\;U\left(\theta_{ij}\right),$$

where ${\vec{r}}_{ij}$ is the vector pointing from molecule *i* to *j* in the body frame of molecule *i*, $r_{ij}=\left|{\vec{r}}_{ij}\right|$, and $\theta_{ij}$ is the angle of this vector in the same frame. The factor 1/2 normalizes the potential so that the maximum contribution from both molecules is 2. The orientational term uses a three-petal Rose function:

(5) $$U\left(\theta_{ij}\right)=a_{2}\sin^{2}\left(3\theta_{ij}\right)+a_{1}\sin\left(3\theta_{ij}\right),$$

where $a_{1}$ and $a_{2}$ control the angular shape of the potential. In Cartesian coordinates of the molecule *j* relative to *i*, this reads:

(6) $$U_{HB}\left({\vec{r}}_{ij}\right)=\frac{\epsilon_{HB}}{2}s\left(r_{ij}\right)\left[a_{2}\frac{{\left(3x_{ij}^{2}y_{ij}-y_{ij}^{3}\right)}^{2}}{r_{ij}^{6}}+a_{1}\frac{3x_{ij}^{2}y_{ij}-y_{ij}^{3}}{r_{ij}^{3}}\right],$$

with $x_{ij}$ and $y_{ij}$ the Cartesian components of ${\vec{r}}_{ij}$ in the body frame of molecule *i*. The distance-dependent term is modeled using a symmetric double-sided cubic switching function:

$$s\left(r_{ij}\right)=\left\{\begin{array}{cc} 0, & r_{ij}<r_{l},\\ \frac{\left(r_{l}+2r_{ij}-3r_{HB}\right){\left(r_{l}-r_{ij}\right)}^{2}}{{\left(r_{l}-r_{HB}\right)}^{3}}, & r_{l}\leq r_{ij}<r_{HB},\\ \frac{\left(r_{u}+2r_{ij}-3r_{HB}\right){\left(r_{u}-r_{ij}\right)}^{2}}{{\left(r_{u}-r_{HB}\right)}^{3}}, & r_{HB}\leq r_{ij}<r_{u},\\ 0, & r_{u}\leq r_{ij}, \end{array}\right.$$

where $r_{HB}$ is the hydrogen bond distance, $r_{l}$ and $r_{u}$ are the lower and upper bounds of the bond, and $r_{FWHM}=|r_{HB}-r_{l}\left|=\right|r_{HB}-r_{u}|$ defines the full width at half maximum of the peak. Parameters were chosen so that the Rose water model reproduces key features of the Mercedes–Benz (MB) water model [52,53]. The MB parametrization uses:

$$\epsilon_{LJ}=0.1,\;\sigma_{LJ}=0.7,\;\epsilon_{HB}=1,\;r_{HB}=1,\;r_{FWHM}=0.2,\;a_{1}=0.6,\;a_{2}=-0.4.$$

A second “real” parametrization is also employed, differing only in the LJ parameters:

$$\epsilon_{LJ}=0.2,\;\sigma_{LJ}=0.890899,$$

so that the minimum of the LJ potential coincides with the hydrogen bond length and its energetic contribution is larger than in the MB parametrization.

## 3. Theory

### 3.1. Monte Carlo Simulations

Monte Carlo simulations were carried out in different ensembles (NVT, NpT, and μVT) using the Metropolis algorithm to calculate the properties of the Rose water model. Periodic boundary conditions combined with the minimum image convention were employed to minimize finite-size effects. In each Monte Carlo step, one molecule was randomly chosen for translational displacement and another for rotational displacement, such that on average every particle underwent one translation and one rotation per simulation cycle. In isothermal-isobaric ensemble attempts to change volume were also made and in grand canonical ensemble attempts to exchange particles were made.

All simulations were initialized from random particle configurations and equilibrated for $10^{6}$ cycles. Following equilibration, production runs were performed consisting of 20 independent series, each of length $10^{6}$ cycles. The system size was fixed at 200 Rose particles. During the sampling phase, structural and thermodynamic properties were evaluated.

The pseudo-diffusion coefficient was determined from the mean-square displacement (MSD) according to

(7) $$D^{*}=\lim_{n\to \infty }\frac{\langle \Delta r{\left(n\right)}^{2}\rangle }{n},$$

with

(8) $$\langle \Delta r{\left(n\right)}^{2}\rangle =\langle {\left[\vec{r}\left(n\right)-{\vec{r}}_{0}\right]}^{2}\rangle ,$$

where ${\vec{r}}_{0}$ denotes the initial particle position and *n* the number of Monte Carlo cycles. The MSD was averaged over all particles in the system. For simple fluids, $D^{*}$ decreases monotonically with increasing density at constant temperature.

Structural anomalies were quantified using translational and orientational order parameters. The translational order parameter was defined as

(9) $$t=\rho^{1/2}\int_{0}^{r_{c}}\left|g\left(r\right)-1\right|\;dr,$$

where g(r) is the radial distribution function, ρ is the number density, and the cutoff distance $r_{c}$ was chosen as half the simulation box length. This parameter characterizes pair correlations and, for normal fluids, increases monotonically with density.

Orientational ordering was examined through the three-fold ($q_{3}$) and six-fold ($q_{6}$) orientational order parameters. In general,

(10) $$q_{l}=\frac{1}{N}\sum_{j=1}^{N}\left|q_{l}^{j}\right|,$$

where $q_{l}^{j}$ for a particle *j* is given by

(11) $$q_{l}^{j}=\frac{1}{n_{j}}\sum_{k}\exp\;\left(il\theta_{jk}\right).$$

Here, $n_{j}$ is the number of neighbors in the first coordination shell of particle *j*, $\theta_{jk}$ is the angle between the vector connecting particles *j* and *k* and the horizontal axis, and *l* specifies the symmetry (l=3 or 6). The first coordination shell was defined as all particles within a distance of 1.2, corresponding to the first minimum following the hydrogen-bonding peak in the Rose-particle radial distribution function.

Pair entropy, which represents the dominant contribution to excess entropy [17,18,69,70,71,72,73,74], was also evaluated. Excess entropy can be expressed as a multiparticle correlation expansion,

(12) $$s_{e}=s_{2}+s_{3}+\ldots +s_{n},$$

where $s_{n}$ denotes the *n*-particle correlation contribution. The pair entropy contribution, $s_{2}$, was computed from

(13) $$s_{2}=-\pi \rho \int_{0}^{L/2}\left[g(r)\ln g(r)-g(r)+1\right]r\;dr,$$

with *L* denoting the simulation box length.

### 3.2. Molecular Dynamics Simulations

Molecular dynamics (MD) simulations were also employed to calculate the properties of the Rose water model, which were subsequently used in further analyses. The simulations were performed with an in-house MD code [75], originally developed for the MB water model but modified in this work to implement the Rose water interaction potential. All simulations were conducted in the NPT ensemble. To mimic macroscopic conditions, periodic boundary conditions and the minimum image convention were applied.

Trajectory integration was carried out using the Velocity Verlet algorithm [76] with a time step of 0.001. To convert from reduced units to SI units of time, the following relation can be used:

$$t^{*}=t\sqrt{\frac{\epsilon_{HB}}{mr_{HB}^{2}}},$$

where *m* is mass of the molecule. Each system was equilibrated for $10^{5}$ steps, followed by a production run consisting of 20 independent series, each of $10^{5}$ steps. The system contained 200 Rose water molecules, initialized from a random non-overlapping configuration.

During equilibration, a simple velocity rescaling thermostat was applied, while the sampling phase employed stochastic velocity rescaling [77]. Pressure control was performed using the Berendsen barostat [78] during equilibration and the stochastic cell rescaling method [79] during sampling. The thermostat and barostat coupling constants were set to 0.01 and 0.1, respectively.

Thermodynamic, structural, and dynamic properties were evaluated using standard relations. Angular distributions were obtained by counting molecular orientations at different positions and representing them as histograms.

### 3.3. Thermodynamic Perturbation Theory

We applied Wertheim’s first-order thermodynamic perturbation theory (TPT) [80,81,82] to describe the thermodynamic properties of the Rose water model. The central quantity in TPT is the Helmholtz free energy. For the Rose water molecule, the Helmholtz free energy *A* is decomposed into a Lennard–Jones (LJ) contribution and a hydrogen-bonding contribution:

(14) $$\frac{A}{Nk_{B}T}=\frac{A_{LJ}}{Nk_{B}T}+\frac{A_{HB}}{Nk_{B}T},$$

where *N* is the number of particles, $k_{B}$ the Boltzmann constant, and *T* the temperature.

The LJ contribution was evaluated using Barker–Henderson perturbation theory [83], in which the system of hard disks (HD) serves as a reference and the LJ potential is treated as a perturbation:

(15) $$\frac{A_{LJ}}{Nk_{B}T}=\frac{A_{HD}}{Nk_{B}T}+\frac{\rho }{2k_{B}T}\int_{\sigma_{LJ}}^{\infty }g_{HD}\left(r,\eta \right)\;u_{LJ}\left(r\right)\;d\vec{r},$$

where $A_{HD}$ is the Helmholtz free energy of a hard disk system, $g_{HD}$ is the radial distribution function of hard disks, η is the packing fraction, and $\sigma_{LJ}$ is the LJ contact distance [83,84].

The hydrogen-bonding contribution was calculated as

(16) $$\frac{A_{HB}}{Nk_{B}T}=3\left(\ln x-\frac{x}{2}+\frac{1}{2}\right),$$

where *x* is the fraction of nonbonded sites per arm [80,81,82]. The mass-action law gives *x* as

(17) $$x=\frac{1}{1+3\rho x\Delta },$$

with Δ defined by

(18) $$\Delta =2\pi \int g_{LJ}\left(r,\rho \right)\;{\bar{f}}_{HB}\left(r\right)\;r\;dr.$$

Here, $g_{LJ}\left(r,\rho \right)$ is the radial distribution function of LJ disks obtained from the Percus–Yevick integral equation, and ${\bar{f}}_{HB}\left(r\right)$ is the orientationally averaged Mayer function of the hydrogen-bonding potential:

(19) $${\bar{f}}_{HB}\left(r\right)={\langle \exp\;\left[-\beta U_{HB}\left(r,\theta_{1},\theta_{2}\right)\right]\rangle }_{\theta_{1},\theta_{2}}-1,$$

where $U_{HB}\left(r,\theta_{1},\theta_{2}\right)$ denotes the hydrogen-bonding potential of a single bonding site. Once the Helmholtz free energy is determined, other thermodynamic properties follow from standard thermodynamic relations [85].

In addition to standard TPT, we employed the improved TPT formulation [86]. In this approach, the effective density $\rho^{\mathrm{ef}}$ replaces the actual density ρ and is defined as

(20) $$\rho^{\mathrm{ef}}=\frac{1}{V^{\mathrm{ef}}},$$

where the effective volume $V^{\mathrm{ef}}$ is given by

(21) $$V^{\mathrm{ef}}=\frac{1}{\rho }-\frac{\bar{n}V^{\prime }}{2}.$$

The quantity $V^{\prime }$ is the excluded volume associated with hydrogen-bond formation, and $\bar{n}$ is the average number of hydrogen bonds per molecule. Since each molecule has three bonding arms and the probability of a site being bonded is (1−x), the average number of bonds per molecule is

(22) $$\bar{n}=3\left(1-x\right).$$

The inaccessible volume $V^{\prime }$ was obtained by simple geometric considerations. Assuming the hydrogen-bond length equals 1 and approximating the LJ core of disks with the hard-disk diameter *d* from Barker–Henderson perturbation theory, $V^{\prime }$ is given by

(23) $$V^{\prime }=\sqrt{d^{2}-\frac{1}{4}}-\frac{\pi d^{2}}{4}.$$

The equations for $\rho^{\mathrm{ef}}$ and *x* were solved iteratively to ensure self-consistency in the improved TPT framework.

In addition to the TPT formulations for pure Rose water, we also applied TPT to describe solvation. The approach follows Wertheim’s thermodynamic perturbation theory [80,81,82,87,88,89], previously adapted for the Mercedes–Benz water model [90]. In the solvation case, the Helmholtz free energy is again written as the sum of Lennard–Jones (LJ) and hydrogen-bonding contributions,

(24) $$\frac{A}{Nk_{B}T}=\frac{A_{LJ}}{Nk_{B}T}+\frac{A_{HB}}{Nk_{B}T},$$

but now the LJ contribution corresponds to a binary mixture of water and solute particles.

The LJ part of the free energy was evaluated using Barker–Henderson perturbation theory [91], with the hard-disk mixture as the reference system:

(25) $$\frac{A_{LJ}}{Nk_{B}T}=\frac{A_{HD}}{Nk_{B}T}+2\pi \rho x_{1}x_{2}d_{12}^{2}g_{12}^{0}\left(d_{12}\right)\;\left(d_{12}-D_{12}\right)+\pi \rho \beta \sum_{kl}x_{k}x_{l}\int_{\sigma_{kl}}^{\infty }g_{kl}^{0}\left(r\right)\;u_{LJ}^{kl}\left(r\right)\;d\vec{r},$$

where $x_{1}$ and $x_{2}$ are mole fractions of water and solute, respectively, ρ is the total number density, $u_{LJ}^{kl}$ are LJ pair potentials, and $g_{kl}^{0}\left(r\right)$ are the pair distribution functions of the reference hard-disk mixture, obtained from the Percus–Yevick integral equation. The quantity $D_{ij}$ is defined as

(26) $$D_{ij}=\int_{0}^{\sigma_{LJ}^{ij}}\left[1-\exp(-\beta u_{LJ}^{ij}\left(r\right))\right]\;dr,$$

and the effective contact distance is given by $d_{12}=\left(D_{11}+D_{22}\right)/2$.

The Helmholtz free energy of the hard-disk mixture was evaluated as [90]

(27) $$\frac{A_{HD}-A_{id}}{Nk_{B}T}=s\left(-\frac{7b}{8}\eta -\left(\frac{7}{8}-\frac{b}{4}\right)\ln\left(1-\eta \right)+\frac{9(1+b)\eta }{8(1-\eta )}\right),$$

with packing fraction $\eta =\left(\pi /4\right)\rho \sum_{i}x_{i}D_{ii}^{2}$,

(28) $$s=\frac{\pi }{4s_{\mathrm{mix}}}\sum_{i}\sum_{j}x_{i}x_{j}D_{ij}^{2},\;s_{\mathrm{mix}}=\frac{\pi }{4}\sum_{i}x_{i}D_{ii}^{2},$$

and parameter *b* given by

(29) $$b=\frac{C_{\mathrm{mix}}^{HD}}{2s}-2\left(\frac{4}{3}-\frac{\sqrt{3}}{\pi }\right),$$

where

(30) $$C_{\mathrm{mix}}^{HD}=\frac{\pi }{3}\left(a_{11}x_{1}^{3}D_{11}^{4}+a_{12}x_{1}^{2}x_{2}D_{12}^{4}+a_{21}x_{1}x_{2}^{2}D_{21}^{4}+a_{22}x_{2}^{3}D_{22}^{4}\right).$$

The coefficients are given by [90]

(31) $$a_{11}=a_{22}=\pi -\frac{3^{3/2}}{4},$$

(32) $$a_{12}=3\left(\pi +2\left(\frac{D_{11}^{2}}{D_{12}^{2}}-1\right)\arccos\left(\frac{D_{11}}{2D_{12}}\right)-\frac{D_{11}^{2}}{2D_{12}^{2}}\left(1+\frac{D_{11}^{2}}{2D_{12}^{2}}\right)\sqrt{\frac{4D_{12}^{2}}{D_{11}^{2}}-1}\right),$$

with $a_{21}$ obtained analogously by exchanging $D_{11}$ with $D_{22}$.

The hydrogen-bonding contribution for the mixture is [90,92,93]

(33) $$\frac{A_{HB}}{Nk_{B}T}=3x_{1}\left(\ln y-\frac{y}{2}+\frac{1}{2}\right),$$

where *y* is the fraction of unbonded water arms, obtained from the mass-action equation

(34) $$y=\frac{1}{1+3\rho_{1}y\Delta },$$

with $\rho_{1}$ the number density of water molecules and

(35) $$\Delta =2\pi \int g_{11}^{LJ}\left(r\right)\;{\bar{f}}_{HB}\left(r\right)\;r\;dr.$$

Here, ${\bar{f}}_{HB}\left(r\right)$ is the orientationally averaged Mayer function of the hydrogen-bond potential for one interaction site, and $g_{11}^{LJ}\left(r\right)$ is the LJ pair distribution function of the mixture, evaluated using the Percus–Yevick equation.

From the Helmholtz free energy, other thermodynamic properties were obtained by standard relations [85]. For instance, the solvation chemical potential of the solute follows as

(36) $$\mu_{2}=\Delta G={\left(\frac{\partial G}{\partial N_{2}}\right)}_{N_{1},p,T}={\left(\frac{\partial A}{\partial N_{2}}\right)}_{N_{1},V,T},$$

with $N_{1}$ and $N_{2}$ denoting the numbers of water and solute particles, respectively.

### 3.4. Integral Equation Theory

We applied Wertheim’s integral equation theory (IET) to study the Rose water model [80,81]. In this approach, an orientationally averaged version of the multidensity Ornstein–Zernike (OZ) equation was solved together with the polymer Percus–Yevick (PPY) closure [80,81,83,94,95]. To make the IET applicable, one additional approximation was introduced compared to MC simulations: the bonding arms of the molecules were not fixed in space.

The multidensity OZ equation used here has the form

(37) $$\hat{h}\left(k\right)=\hat{c}\left(k\right)+\hat{c}\left(k\right)\rho \hat{h}\left(k\right),$$

where $\hat{h}\left(k\right)$ and $\hat{c}\left(k\right)$ are matrices whose elements are Fourier transforms of the partial correlation functions $h_{ij}\left(r\right)$ and $c_{ij}\left(r\right)$. Instead of Wertheim’s original density parameters, a partial density matrix was introduced. In addition, the ideal network approximation was adopted, meaning that correlations responsible for ring structures were neglected [94,95]. Because the bonding arms are equivalent, the correlation matrices reduce to 2×2 form:

(38) $$\hat{w}\left(k\right)=\left(\begin{array}{cc} {\hat{w}}_{00}\left(k\right) & {\hat{w}}_{01}\left(k\right)\\ {\hat{w}}_{10}\left(k\right) & {\hat{w}}_{11}\left(k\right) \end{array}\right),$$

with

(39) $$\rho =\left(\begin{array}{cc} \rho & 3\rho \\ 3\rho & 6\rho \end{array}\right),$$

where ρ is the average number density. The prefactors 3 and 6 arise from this dimensionality reduction.

To solve the OZ equation, the PPY closure was applied [80,81]:

(40) $$c_{ij}\left(r\right)=f_{LJ}\left(r\right)\left[t_{ij}\left(r\right)+\delta_{i0}\delta_{j0}\right]+\delta_{i1}\delta_{j1}x^{2}{\bar{f}}_{HB}\left(r\right)e_{LJ}\left(r\right)\;\left[t_{00}\left(r\right)+1\right],$$

where t(r)=h(r)−c(r), $f_{LJ}\left(r\right)=e_{LJ}\left(r\right)-1$, $e_{LJ}\left(r\right)=\exp\left(-\beta U_{LJ}\left(r\right)\right)$, and *x* is the fraction of particles not bonded at one arm. The orientationally averaged Mayer function of the hydrogen-bond potential is

(41) $${\bar{f}}_{HB}\left(r\right)={\langle \exp\;\left(-\beta U_{HB}\left(r,\theta_{1},\theta_{2}\right)\right)\rangle }_{\theta_{1},\theta_{2}}-1.$$

The mass–action relation gives *x* as

(42) $$x=\frac{1}{1+3\rho x\Delta },$$

with

(43) $$\Delta =2\pi \int g_{00}\left(r,\rho \right){\bar{f}}_{HB}\left(r\right)\;rdr.$$

The total radial distribution function is reconstructed as

(44) $$g\left(r\right)=g_{00}\left(r\right)+3g_{01}\left(r\right)+3g_{10}\left(r\right)+9g_{11}\left(r\right),$$

where $g_{ij}\left(r\right)=h_{ij}\left(r\right)+\delta_{i0}\delta_{j0}$ [83]. The OZ and PPY equations were solved iteratively, and the Talman method was used to perform Fourier–Bessel transforms [96].

Once g(r) was obtained, thermodynamic quantities followed from standard relations.

The pressure was obtained from the virial equation [80,81].

To evaluate properties at constant pressure, the density corresponding to the target pressure was determined using the bisection method. This enabled the calculation of thermodynamic observables at the corrected density.

### 3.5. Orientation-Dependent Integral Equation Theory

Here, we outline the orientation-dependent formulation of Wertheim’s multidensity Ornstein–Zernike (OZ) approach, which we employed for the description of the Rose water model. The theoretical framework is largely based on the version previously applied to the MB model [97], although several modifications were required in order to adapt it to the Rose model.

For a system of Rose model particles, Wertheim’s multidensity OZ relation is expressed as [97,98]:

(45) $$\begin{array}{cc} h_{\alpha \beta }^{\left(W\right)}\left(r_{12},\theta_{1},\theta_{2}\right) & =c_{\alpha \beta }^{\left(W\right)}\left(r_{12},\theta_{1},\theta_{2}\right)\\ \; & \;+\frac{1}{2\pi }\int \sum_{\mu \nu }c_{\alpha \mu }^{\left(W\right)}\left(r_{13},\theta_{1},\theta_{3}\right)\sigma_{\mu \nu }h_{\nu \beta }^{\left(W\right)}\left(r_{32},\theta_{3},\theta_{2}\right)\;dr_{3}\;d\theta_{3}, \end{array}$$

where the superscript *W* refers to Wertheim’s formalism, indices α, β, μ, and ν denote bonding sites on the Rose particle, and $\sigma_{\mu \nu }$ are Wertheim density parameters [80,81].

Direct numerical treatment of this equation is impractical. To make it tractable, the correlation functions are expanded in orthogonal functions [97,98,99]:

(46) $$z_{\alpha \beta }^{\left(W\right)}\left(r_{12},\theta_{1},\theta_{2}\right)=\sum_{m,j=-L}^{L}z_{\alpha \beta }^{\left(W\right)}\left(r,m,j\right)\exp\left[i\left(m\theta_{1}+j\theta_{2}\right)\right].$$

Although the summation is formally infinite, only a finite number of coefficients is typically sufficient, with L<9 in practice.

Fourier transformation provides a link between reciprocal and real-space correlation functions:

(47) $${\hat{z}}_{\alpha \beta }^{\left(W\right)}\left(k,m,j\right)=2\pi i^{m+j}\int_{0}^{\infty }z_{\alpha \beta }^{\left(W\right)}\left(r,m,j\right)J_{m+j}\left(kr\right)\;r\;dr,$$

(48) $$z_{\alpha \beta }^{\left(W\right)}\left(r,m,j\right)=\frac{1}{2\pi i^{m+j}}\int_{0}^{\infty }{\hat{z}}_{\alpha \beta }^{\left(W\right)}\left(k,m,j\right)J_{m+j}\left(kr\right)\;k\;dk,$$

with $J_{p}$ denoting the Bessel function of the first kind of order *p*.

After transforming Equation (45) to Fourier space and integrating over the angular variable $\theta_{3}$, one obtains

(49) $${\hat{h}}_{\alpha \beta }\left(k,m,j\right)={\hat{c}}_{\alpha \beta }\left(k,m,j\right)+\sum_{p=-L}^{L}\sum_{\mu \nu }{\hat{c}}_{\alpha \mu }\left(k,m,-p\right)\;\rho_{\mu \nu }\;{\hat{h}}_{\nu \beta }\left(k,p,j\right).$$

To avoid divergences at low temperature, we employed partial correlation functions $z_{\alpha \beta }$ rather than Wertheim’s original $z_{\alpha \beta }^{\left(W\right)}$. These quantities are related by

(50) $$\rho \;z_{\alpha \beta }\;\rho =\sigma_{ABC-\alpha }\;z_{\alpha \beta }^{\left(W\right)}\;\sigma_{ABC-\beta },$$

where ρ is the number density of Rose particles.

Equation (49) can be written in matrix form as

(51) $$\hat{t}={\hat{c}}^{-}\;\rho \;\left[\hat{t}+\hat{c}\right],$$

which can be solved for $\hat{t}$ to yield

(52) $$\hat{t}={\left(I-{\hat{c}}^{-}\rho \right)}^{-1}{\hat{c}}^{-}\rho \hat{c},$$

with $\hat{t}=\hat{h}-\hat{c}$.

To solve the OZ equations, an additional closure is necessary. Here, we adopt the polymer soft mean-spherical approximation (PSMSA) [100].

The closure that was used has follows: form

(53) $$\begin{array}{cc} c_{\alpha ,\beta }^{\left(W\right)}\left(r,\theta_{1},\theta_{2}\right) & =f_{0}\left(r\right)y_{\alpha ,\beta }^{\left(W\right)}\left(r,\theta_{1},\theta_{2}\right)\\ \; & \;+e_{LJ}\left(r\right)\sum_{D\in \alpha }\sum_{E\in \beta }f_{DE}\left(r,\theta_{1},\theta_{2}\right)y_{\alpha -D,\beta -E}^{\left(W\right)}\left(r,\theta_{1},\theta_{2}\right)\left(1-\delta_{\alpha ,0}\right)\left(1-\delta_{\beta ,0}\right), \end{array}$$

with $e_{LJ}\left(r\right)=\exp\left(-U_{LJ}\left(r\right)/k_{B}T\right)$, $f_{0}\left(r\right)=\exp\left(-U_{0}\left(r\right)/k_{B}T\right)-1$, and $\delta_{\alpha ,0}$ the Kronecker delta.

The coupled equations were solved by iterative methods, using Talman’s method [96] for the Bessel–Fourier transforms. Orientationally averaged and angle-resolved pair distribution functions follow from the correlation functions as

(54) $$g\left(r\right)=\sum_{\alpha ,\beta }h_{\alpha ,\beta }\left(r,0,0\right)+1,$$

(55) $$g\left(r,m,j\right)=\sum_{\alpha ,\beta }\left(h_{\alpha ,\beta }\left(r,m,j\right)+\delta_{\alpha ,0}\delta_{\beta ,0}\delta_{m,0}\delta_{j,0}\right),$$

(56) $$g\left(r,\theta_{1},\theta_{2}\right)=\sum_{m,j=-L}^{L}g\left(r,m,j\right)\exp\left[i\left(m\theta_{1}+j\theta_{2}\right)\right].$$

Once the pair distribution functions are available, standard statistical mechanics allows the evaluation of other thermodynamic functions.

The orientation-dependent integral equation theory was further extended to treat systems containing a solute dispersed in the Rose water fluid. The general framework is the same as for the pure solvent case, with the difference that additional cross-correlation functions between solvent and solute must be considered. The formalism used here follows the approach previously applied to solvation in the Mercedes–Benz water model [101], but adapted to the present Rose water parametrization [58].

As in the solvent-only case, correlation functions are expanded into a complete orthogonal basis [97,98,99],

(57) $$z_{\alpha \beta }\left(r_{12},\theta_{1},\theta_{2}\right)=\sum_{m,j=-L}^{L}z_{\alpha \beta }\left(r,m,j\right)\exp\left[i\left(m\theta_{1}+j\theta_{2}\right)\right],$$

where the truncation index *L* limits the number of harmonics needed (typically L<9). Fourier–Bessel transforms connect real- and *k*-space representations:

(58) $${\hat{z}}_{\alpha \beta }\left(k,m,j\right)=2\pi i^{m+j}\int_{0}^{\infty }z_{\alpha \beta }\left(r,m,j\right)J_{m+j}\left(kr\right)rdr,$$

(59) $$z_{\alpha \beta }\left(r,m,j\right)=\frac{1}{2\pi i^{m+j}}\int_{0}^{\infty }{\hat{z}}_{\alpha \beta }\left(k,m,j\right)J_{m+j}\left(kr\right)kdk,$$

with $J_{p}$ denoting the Bessel function of order *p*.

After angular integration, the multidensity OZ equation takes the form

(60) $${\hat{h}}_{\alpha \beta }\left(k,m,j\right)={\hat{c}}_{\alpha \beta }\left(k,m,j\right)+\sum_{p=-L}^{L}\sum_{\mu \nu }{\hat{c}}_{\alpha \mu }\left(k,m,-p\right)\rho_{\mu \nu }{\hat{h}}_{\nu \beta }\left(k,p,j\right),$$

or, equivalently, in compact notation,

(61) $$\hat{h}=\hat{c}+{\hat{c}}^{-}\rho \hat{h},$$

where ρ is now a block matrix containing both solvent–solvent and solute–solvent densities. The structure of these matrices follows directly from the combinatorics of bonding states, with detailed definitions given in our earlier work [58].

To solve the equations, the polymer soft mean-spherical approximation (PSMSA) [100] was applied. This closure had already shown improved accuracy compared to the polymer Percus–Yevick form in related MB water studies [97], and the same advantage is assumed to hold for the Rose model.

As in the pure water, the direct iteration method was used to solve the coupled set of OZ and closure equations, and Fourier–Bessel transforms were handled using Talman’s method [96].

From the converged solutions, orientationally averaged radial distribution functions were obtained, e.g.,

(62) $$g^{11}\left(r\right)=\sum_{\alpha ,\beta }h_{\alpha ,\beta }^{11}\left(r,0,0\right)+1,\;g^{12}\left(r\right)=\sum_{\alpha }h_{\alpha }^{12}\left(r,0,0\right)+1,\;g^{22}\left(r\right)=h^{22}\left(r,0,0\right)+1.$$

Higher-order coefficients were likewise reconstructed from the harmonic expansions, allowing the final two-particle distribution $g(r,\theta_{1},\theta_{2})$ to be recovered.

Thermodynamic functions such as the internal energy and pressure were then evaluated by applying the same statistical–mechanical relations as in the pure solvent case, but with the inclusion of solute–solvent contributions. Pressure was calculated both via the compressibility route and through the virial expression, and other quantities were derived using standard thermodynamic identities.

### 3.6. Analytical Model

We first provide a short overview of the UD (Urbic Dill) model, which serves as the basis for evaluating the thermodynamic and dynamical behavior of water. A more extensive description of the model is given in [102]. The framework considers a system of *N* water molecules, focusing on the behavior of a single molecule arranged in a hexagonal environment together with its neighbors. Each water molecule is assumed to possess three equivalent interaction arms, representing one-third of the molecule, capable of forming hydrogen bonding. Because all three arms are equivalent, threefold symmetry is explicitly taken into account. Each arm can occupy one of three distinct interaction states: (i) a hydrogen bond (HB), (ii) a van der Waals/Lennard–Jones (LJ) contact, or (iii) a noninteracting state. For each possible state, the corresponding isothermal-isobaric statistical weight can be evaluated.

Hydrogen-Bonded State

When a molecule forms a hydrogen bond, one of its arms is directed toward the center of a neighboring molecule, such that the deviation angle between the arm orientation and the intermolecular axis is less than π/3. The interaction energy of this configuration is given by

(63) $$u_{HB}\left(\theta \right)=-\epsilon_{HB}-\epsilon_{LJ}+k_{s}\theta^{2},\;-\pi /3<\theta <\pi /3,$$

where $\epsilon_{HB}$ denotes the energy parameter of the hydrogen bond, $\epsilon_{LJ}$ represents the energetic contribution from the LJ contact, and $k_{s}$ is the angular spring constant that accounts for the angular dependence of hydrogen bonding. This formulation describes a single hydrogen bond; cooperative effects are incorporated later.

vdW/LJ contact state

In the second possible state, neighboring molecules experience direct van der Waals or LJ interactions without forming a hydrogen bond. The energy associated with this interaction is

(64) $$u_{LJ}=-\epsilon_{LJ}.$$

Noninteracting State

Finally, when two molecules do not interact, their energy is simply

(65) $$u_{0}=0.$$

Partition Function

The isothermal–isobaric partition functions of all interaction states are combined to yield the partition function of a hexagonal cluster of six molecules:

(66) $$Q_{1}={\left(\Delta_{HB}+\Delta_{LJ}+\Delta_{0}\right)}^{6}.$$

This expression assumes independent behavior of molecules within the hexagon. However, hydrogen bonding is also cooperative and extends beyond simple pairwise interactions. To incorporate this, the partition function is modified such that a fully hydrogen-bonded hexagonal configuration is energetically favored:

(67) $$Q_{1}={\left(\Delta_{HB}+\Delta_{LJ}+\Delta_{0}\right)}^{6}-\Delta_{HB}^{6}+\delta \Delta_{s}^{6},$$

with $\delta =\exp(-\beta \epsilon_{c})$ being the Boltzmann factor corresponding to the cooperative energy $\epsilon_{c}$. Here, $\Delta_{s}$ differs from $\Delta_{HB}$ in that it employs $v_{s}$ instead of $v_{HB}$, reflecting the case where all molecules are connected via hydrogen bonds to form a perfect hexagonal lattice. The partition function for a system of *N* molecules then becomes $Q=Q_{1}^{N/6}$. Various thermodynamic properties can be derived from this partition function. Importantly, the influence of external conditions on the system is not imposed a priori but emerges from the partition function itself. The four relevant interaction states i=1 (HB), 2 (LJ), 3 (0), and 4 (s) are described by their populations:

(68) $$f_{i}=\frac{\partial \log Q_{1}}{\partial \log\Delta_{i}^{6}}.$$

Other thermodynamic properties are determined using standard statistical-mechanical relations, as outlined in [102,103].

The description of dynamical properties builds directly on the UD model. For a comprehensive discussion, see [104]; here we provide a short summary. The essential idea is that molecular motion in the liquid and gaseous states can be described as a random walk, where diffusion in two dimensions is expressed as

(69) $$D\propto \lambda^{2}\nu ,$$

with λ being the step length and ν the step frequency. The overall diffusion coefficient of water is then represented as a weighted sum of state-dependent contributions:

(70) $$D=T\sum f_{i}D_{i},$$

where $D_{i}=\lambda_{i}^{2}\nu_{i}$ corresponds to the diffusion coefficient of a molecule in a given state (*i* = HB, LJ, 0, s). The step lengths are estimated as follows: $\lambda_{HB}=\lambda_{s}=r_{HB}$ for HB and solid states, $\lambda_{LJ}=\sigma_{LJ}$ for the LJ state, and $\lambda_{0}=\sqrt{v_{0}}$ for the noninteracting state, where $v_{0}$ is the average free volume available in that state. The step frequencies are determined by the Boltzmann factor of the mean energy of each state:

(71) $$\nu_{i}=C\exp\left(\beta \langle u_{i}\rangle \right),$$

with *C* being a constant that serves only as a unit factor.

#### Structural Model

To complement the thermodynamic description, we constructed a structural model of water that employs the probabilities obtained from the UD model to generate spatial molecular arrangements. The model is inspired by snowflake growth, producing a disordered hexagonal hydrogen-bonding network resembling liquid water and monolayer structures [24,34].

As in the UD model, each molecule possesses three arms, which can form hydrogen-bond (HB), Lennard–Jones (LJ), or noninteracting contacts. Cooperative hydrogen bonding is implicitly included by adding the *s*-state population to the HB probability, such that the probabilities for new interactions are

$$P_{HB}=f_{HB}+f_{s},\;P_{LJ}=f_{LJ},\;P_{0}=f_{0}.$$

The construction starts from a central molecule at (0,0) with orientation θ=0. For each arm k∈{0,1,2} of molecule *i*, the coordinates of the new molecule are generated as

(72) $$\begin{array}{cc} x_{i,k} & =x_{i-1}+\cos\left(\theta_{i-1}+kq+Z_{\theta ,j}\right)\left(r_{j}+Z_{r,j}\right),\\ y_{i,k} & =y_{i-1}+\sin\left(\theta_{i-1}+kq+Z_{\theta ,j}\right)\left(r_{j}+Z_{r,j}\right),\\ \theta_{i,k} & =\theta_{i-1}+kq+Z_{\theta ,j}+\pi , \end{array}$$

where $q=120^{\circ }$, $r_{j}$ is the interaction length, and $Z_{r,j}$, $Z_{\theta ,j}$ are random displacements accounting for thermal motion. Interaction distances are

$$r_{HB}=r_{HB},\;r_{LJ}=\sigma_{LJ}2^{1/6},\;r_{0}=\sqrt{T/p}+r_{LJ}.$$

Thermal fluctuations are sampled from distributions depending on interaction type:

(73) $$\begin{array}{cc} Z_{r,HB} & ∼N\;\left(0,{\left(\sqrt{\frac{k_{B}T}{m_{1}k_{HB}}}A\right)}^{2}\right),\;Z_{\theta ,HB}∼N\;\left(0,{\left(\sqrt{\frac{k_{B}T}{m_{3}k_{HB}}}A\right)}^{2}\right),\\ Z_{r,LJ} & ∼N\;\left(0,{\left(\sqrt{\frac{k_{B}T}{m_{1}k_{LJ}}}A\right)}^{2}\right),\;Z_{\theta ,LJ}∼U\;\left(-\frac{\pi }{3},\frac{\pi }{3}\right),\\ Z_{r,0} & ∼N\;\left(0,{\left(\sqrt{\frac{k_{B}T}{m_{1}k_{0}}}A\right)}^{2}\right),\;Z_{\theta ,0}∼U\;\left(-\frac{\pi }{3},\frac{\pi }{3}\right). \end{array}$$

Here, N and U denote normal and uniform distributions, $k_{j}$ are harmonic spring constants, and *A* is an empirical scaling parameter. Interaction potentials are approximated by harmonic forms $U_{j}=\frac{1}{2}k_{j}x^{2}-\epsilon_{j}$, with $k_{HB}$ from the thermodynamic model, $k_{LJ}$ fitted to the LJ potential, and $k_{0}$ chosen empirically to ensure physically reasonable radial distribution functions.

The algorithm proceeds shell by shell: each molecule in shell *i* contributes two new arms for shell i+1. Molecule overlap is prevented by enforcing a minimum separation $\sigma_{LJ}-0.05$, except near the central molecule where overlaps are allowed with probability $\exp[-k_{LJ}{\left(r-r_{LJ}\right)}^{2}/T]$ to ensure continuity of the radial distribution function. The procedure terminates once either the maximum shell number is reached or no new molecules can be placed.

Statistical averages are obtained by generating a large number of independent snowflakes (typically $10^{5}$ replicas). From these, radial, angular, and spatial distribution functions are computed analogously to simulations. The approach is conceptually similar to Monte Carlo simulations but is computationally far more efficient. All parameters were taken from the thermodynamic model except for the scaling constant *A*, which was tuned so that radial distribution function peak heights matched simulation results.

## 4. Results and Discussion

### 4.1. Pure Water

#### 4.1.1. Thermodynamic Properties

**Figure 1:**
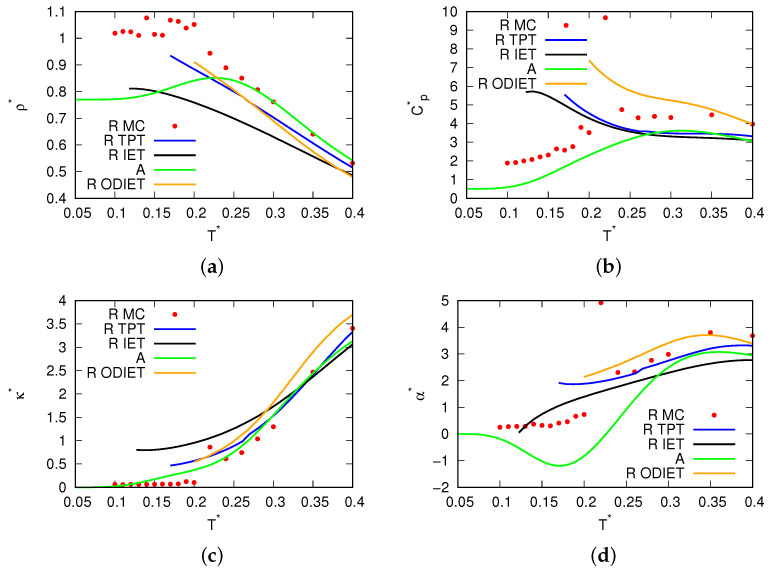
Thermodynamic properties as functions of temperature at pressure 0.19. (**a**) density, (**b**) heat capacity, (**c**) isothermal compressibility, (**d**) thermal expansion coefficient. The plot shows results for analytical (A) and Rose (R) models calculated using Monte Carlo (MC), thermodynamic perturbation theory (TPT), (standard) integral equation theory (IET), and orientation-dependent integral equation theory (ODIET).

Various thermodynamic functions were calculated as functions of temperatures using different methods. The methods used to calculate the functions were Monte Carlo simulations [57], thermodynamic perturbation theory [57], orientation-averaged integral equation theory [57], orientation-dependent integral equation theory [58], and an analytical model [68]. Overall, the trends of the thermodynamic functions predicted by all methods are similar, but there are also some differences. Here, the results of the Monte Carlo simulations are taken as a reference against which the results of the other methods are compared. Let us start with the density. The density maximum in the liquid state is not visible in Figure 1a. The Rose water model with real parameterization also has a solid state with a higher density than the liquid state at pressure 0.19. This is due to the fact that the Rose water model with real parameterization has a quite high probability of occupying the hexagonal interstitial sites. There are two reasons for the increased probability of interstitial site occupation. The first is that the Rose model can form hydrogen half bonds, and the second is that in the real parameterization, the Lennard–Jones contact distance and the length of the hydrogen bonds are the same. Therefore, if a molecule occupies an interstitial site, it can also form half hydrogen bonds with its neighboring molecules, which greatly increases the probability of site occupation. As the temperature increases, the density decreases as the structure of the system becomes increasingly disordered. The methods used here are mostly adapted for the liquid and gaseous states and are therefore less suitable for analyzing the solid state. In Figure 1, it can also be seen that some of the methods do not converge at low temperatures where the solid state occurs. At higher temperatures, the density of the system is most successfully predicted by the analytical model, as the analytical model was parameterized based on the density from the simulations. At lower temperatures, however, the density of the analytical model and the density calculated with MC differ, as the analytical model shows a clearly visible density maximum at a temperature around 0.25. The thermodynamic perturbation theory and the orientation-dependent integral equation theory also predict the density quite well, while the orientation-averaged version of the IET is less successful. Other thermodynamic functions shown in Figure 1 are the heat capacity, the isothermal compressibility, and the coefficient of thermal expansion. Qualitatively, the best results are obtained with the analytical model, since all the thermodynamic functions shown have a similar shape to those calculated with MC, but quantitatively the analytical model is not the most successful. Using the orientation-dependent integral equation theory instead of the orientation-averaged version improves all thermodynamic functions. The thermodynamic perturbation theory predicts the thermodynamic functions quite well, but not as well as the orientation-dependent IET.

#### 4.1.2. Structural Properties

**Figure 2:**
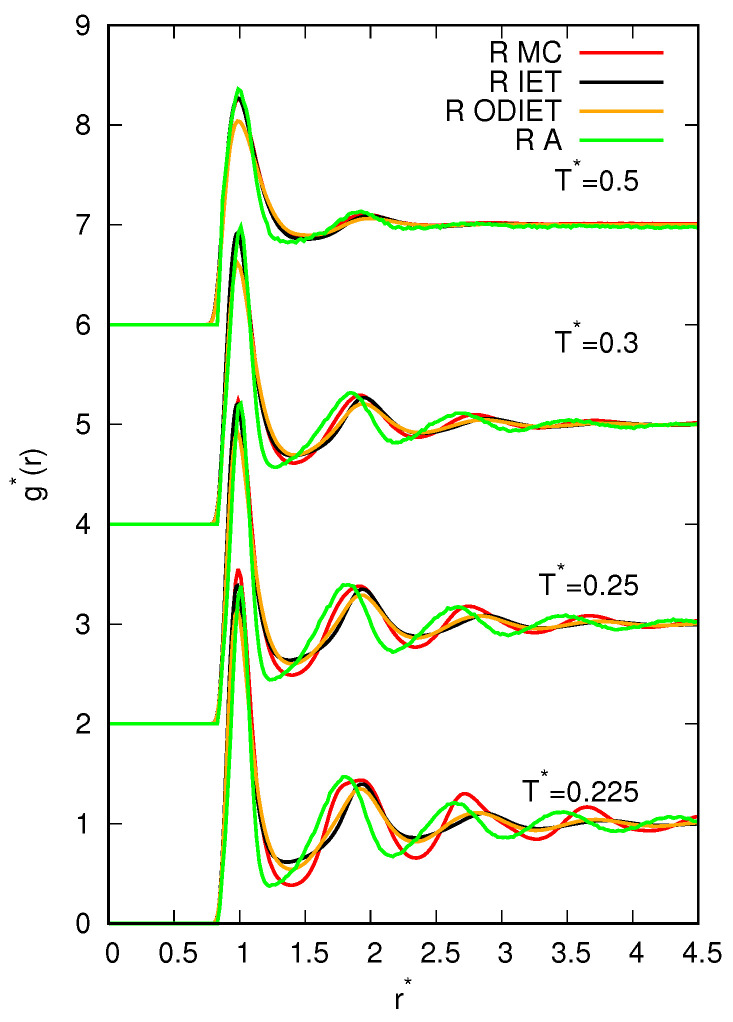
Radial distribution functions at different temperatures and pressure 0.19.

Figure 2 shows the radial distribution functions between the particles of the Rose model at different temperatures [57,58,68]. The agreement between the radial distribution functions calculated with MC and other methods is good and improves with increasing temperature. The main peaks correspond to the typical structures that occur in the system due to hydrogen bonding. Therefore, the height of the peaks decreases with increasing temperature as the structure becomes more disordered. The most obvious difference between the RDFs calculated with MC and any other method is that the peaks obtained with the analytical model are shifted towards closer distances. This is due to the difference in the probability of occupation of the hexagonal interstitial sites when comparing the Rose model and the analytical model. In the Rose model, the occupation of these sites is very favorable, but in the analytical model, the occupation of these sites is not directly included in the model, so it is much less favorable. In Figure 2, the red line (MC) at a temperature of 0.225 has a broader second peak, which can be seen as two merged peaks (the first corresponding to the second shell of the molecules in the hydrogen-bonded structure and the second corresponding to the second shell of the hexagonal interstitial sites). Thus, removing particles from the interstitial sites, the peak shifts towards shorter distances. Nevertheless, the analytical model is very successful in predicting the height of the peaks, as they agree very well with the simulations. The agreement between the RDFs calculated with simulations and both versions of IET is good. The orientation-averaged version of IET predicts the height of the peaks slightly better than the orientation-dependent version. On the other hand, the orientation-dependent version is more successful in predicting the depth of the minima.

**Figure 3:**
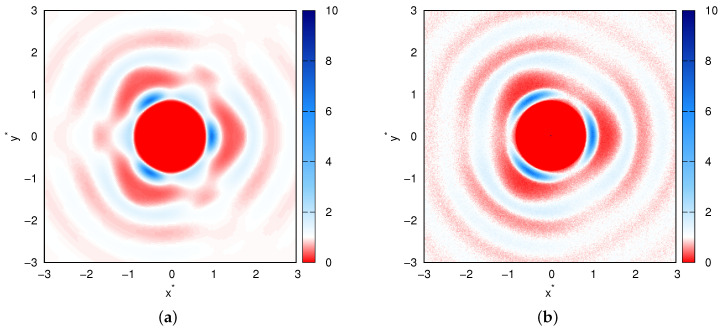
Spatial distribution function between water molecules calculated using: (**a**) ODIET, (**b**) analytical model. Temperature is 0.25 and pressure is 0.19.

With the orientation-dependent IET [58] and the analytical model [68], we can also calculate spatial distribution functions (Figure 3) in a reasonable computational time. The overall shape of the spatial distribution functions calculated with both methods is similar, but there are some significant differences. Triple symmetry can be observed in both spatial distribution functions, and both distributions have alternating higher and lower probability shells. The clearest difference can be seen at the angles 60°, 180°, and 300° from the center of the distributions. In the spatial distribution function calculated by ODIET, the shells are shifted towards larger distances at these angles, whereas this is not the case in the SDF calculated with the analytical model. This is due to the fact that the Rose model used in ODIET has a higher probability of occupying the interstitial site than the analytical model. This is the same effect as shown in Figure 2, but from a different perspective.

### 4.2. Nonpolar Solute in Water

**Figure 4:**
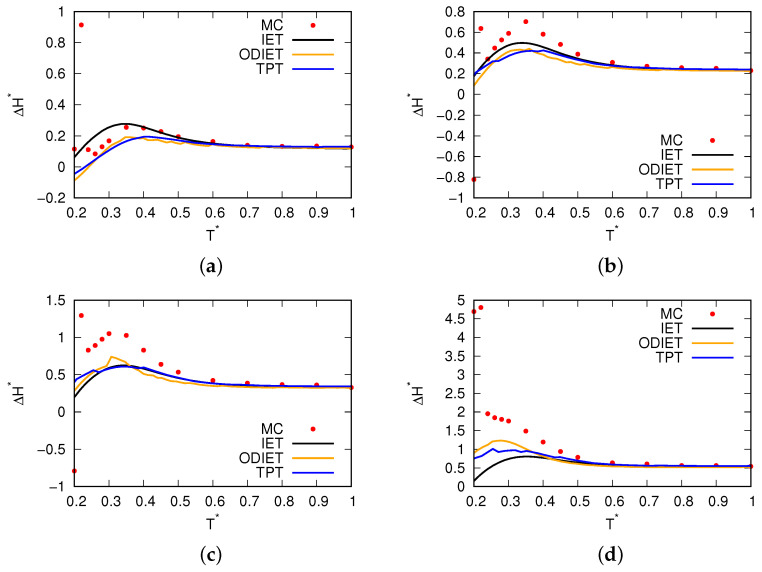
Enthalpy of solvation as function of temperature. Size of nonpolar solute is: (**a**) 0.3, (**b**) 0.7, (**c**) 1.0, (**d**) 1.5, pressure is 0.19.

The enthalpy of solvation of nonpolar solute in Rose water particles calculated using various methods [57,59,60] is shown in Figure 4. If the size of the solute is small (0.3), the enthalpy of solvation has a large spike at low temperatures where the molecules are in the solid state. When the temperature is increased, the enthalpy of solvation decreases and reaches a minimum. This happens because the molecules in a liquid state can better solvate the solute as in the solid state. If the temperature is increased further, the enthalpy increases as the structure of the water becomes more disordered and the solvation of the solute in it becomes less favorable as the hydrogen bonds have to be broken. At higher temperatures, at which most of the hydrogen bonds are already broken, solvation becomes more favorable, as the structure of the hydrogen bonds is no longer disturbed by the solute. For medium solutes (0.7 and 1.0), the general trend of how the enthalpy of solvation changes with temperature is similar to that of small solutes, but they differ at low temperatures. The solvation of medium solutes is much more favorable at low temperatures, as their size is suitable to be inserted into hexagonal interstitial sites and form favorable interactions with neighboring molecules. On the other hand, the insertion of large solutes (1.5) into the structurally ordered system of Rose water particles is extremely unfavorable as it requires the breaking of hydrogen bonds. Therefore, the enthalpy of solvation at low temperature is high for large solutes. When it comes to predicting the enthalpy of solvation using different methods, none of the methods shown in Figure 4 is exceptionally better than the others. At high temperatures, all methods predict the enthalpy perfectly, while at lower temperatures the enthalpy predicted by MC and other methods is slightly different. All methods are able to qualitatively predict the general trend of how enthalpy changes with temperature (local maximum around temperature 0.3), but none are able to quantitatively predict the results of the simulation. Furthermore, none of the methods are able to predict the enthalpy of solvation at low temperatures where a solid state is present. This is to be expected as the methods are not optimized for the solid state.

### 4.3. Anomalies of Rose Water Model

**Figure 5:**
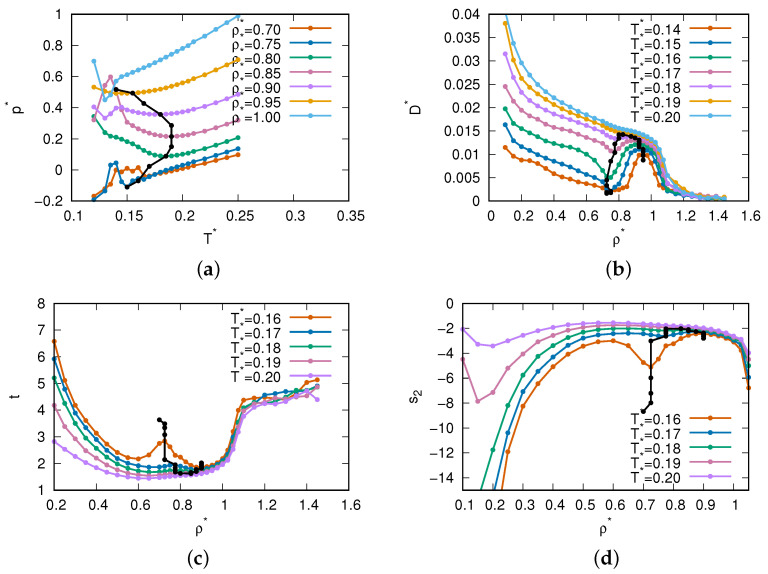
(**a**) Pressure as a function of temperature at different densities. (**b**) pseudo-diffusion coefficient as a function of density at different temperatures. (**c**) translational order parameter as a function of density at different temperatures. (**d**) pair entropy as a function of density at different temperatures. Extrema of functions are connected with black lines. Lines are slightly smoothed using the Savitzky–Golay filter for clearer visualization. For easier visibility, not all isotherms are plotted, while all anomalous points (black dots) are plotted.

MC simulations were used to examine a wider range of conditions to determine where the Rose model exhibits anomalies in various properties [61]. Figure 5 shows how the different anomalies were determined. The density anomaly was determined by locating the minimum of the pressure as a function of temperature at constant density. This minimum appears under the same conditions as the maximum density as a function of temperature at constant pressure. The diffusion of normal fluids decreases with increasing density; therefore, the increase in the diffusion or pseudo-diffusion coefficient with increasing density is considered anomalous. The region in which the pseudo-diffusion coefficient is anomalous was determined by locating local maxima and minima of the coefficient, since diffusion increases with density between these extremes. A similar approach (by locating local extrema) was used to determine anomalous regions for the translational order parameter and the pair entropy. The translational order parameter of normal fluids increases with increasing density, while the pair entropy decreases. The regions in which the opposite is the case are considered anomalous regions.

**Figure 6:**
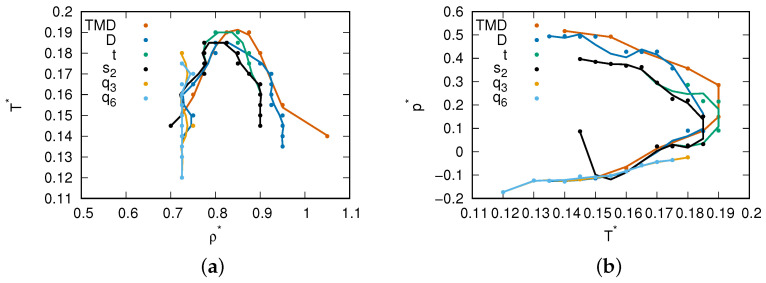
Position of different anomalies (temperature of maximum density (TMD), diffusion (D), translational order (t), pair entropy ($s_{2}$), three-fold ($q_{3}$), and six-fold ($q_{6}$) orientation order parameter) of the Rose model in the temperature-density (**a**) and pressure-temperature (**b**) plane. Trend lines smoothed with the Savitzky–Golay filter are added for easier interpretation.

After locating the regions where different anomalies occur, the hierarchy of anomalies can be determined based on the size of the regions where different anomalies occur. Figure 6 shows the hierarchy of anomalies for the Rose water model with real parameterization. The hierarchy differs from that for real (experimental) water or silica. In Figure 6, the maxima of orientational order parameters is also plotted.

### 4.4. Phase Behaviour of Rose Water Model

#### 4.4.1. Liquid Part of Phase Diagram

**Figure 7:**
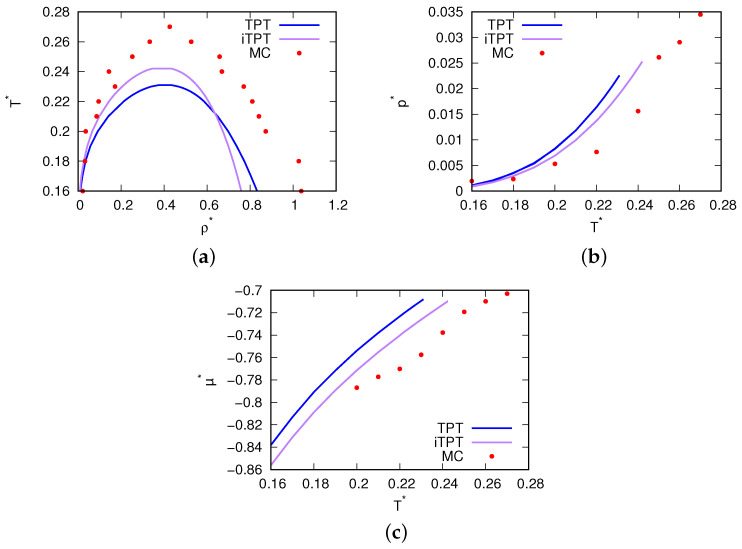
Liquid-vapor equilibrium lines calculated using standard TPT (blue line), improved TPT (purple line), and MC simulations (red points) for real parametrization of the Rose model. (**a**) $T^{*}-\rho^{*}$ liquid–gas equilibrium line, (**b**) $p^{*}-T^{*}$ liquid–gas equilibrium line, and (**c**) $\mu^{*}-T^{*}$ liquid–gas equilibrium line.

To investigate the liquid part of the phase diagram of the Rose water model, Monte Carlo simulations were performed in the grand canonical ensemble [62]. The phase transition between liquid and gaseous phases was determined using density as a function of chemical potential at constant temperature. Liquid-vapor coexistence lines were also calculated using the standard and improved TPT, with the Maxwell construction used to determine the phase transition [62]. The equilibrium lines obtained using all three methods are shown in Figure 7. The critical parameters of the Rose model determined from Monte Carlo simulations with real parameterization for the phase transition between liquid and gas are as follows: $T_{c}^{*}=0.27$, $p_{c}^{*}=0.0345$, $\mu_{c}^{*}=-0.703$, and $\rho_{c}^{*}=0.426$. For comparison, the critical point predicted by the standard TPT is at $T_{c}^{*}=0.231$, $p_{c}^{*}=0.0226$, $\mu_{c}^{*}=-0.708$, and $\rho_{c}^{*}=0.405$, while the improved TPT gives $T_{c}^{*}=0.242$, $p_{c}^{*}=0.0253$, $\mu_{c}^{*}=-0.710$, and $\rho_{c}^{*}=0.390$.

**Figure 8:**
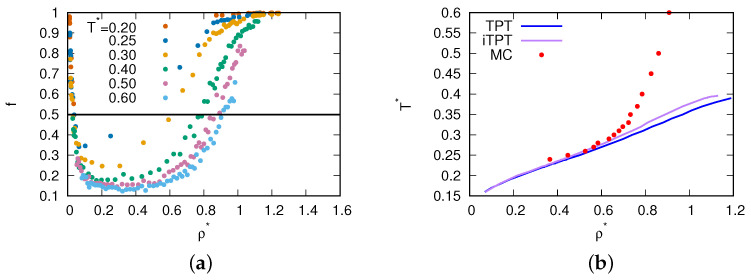
(**a**) Density dependence of the fraction of the molecules belonging to the biggest cluster; results are obtained using Monte Carlo simulations. Different colors represent different temperatures. (**b**) Percolation line from MC and TPT. Results calculated using MC are plotted with red points; results from the standard TPT with the blue line; and results from the improved TPT with the purple line.

The percolation line was also determined for the Rose model with real parameterization [62]. A cluster analysis was performed using MC to check the proportion of molecules connected to the largest cluster by hydrogen bonds. Figure 8a shows how the fraction of molecules connected to the largest cluster changes with density. At low temperatures, the fraction of molecules increases abruptly with density, while at higher temperatures the fraction increases gradually. The percolation line was determined as the density at which the fraction of molecules belonging to the largest cluster is at least 0.5. The percolation line was also determined with TPT as the density at which the fraction of molecules not bonded at a particular arm is 0.5. Figure 8b shows the percolation lines determined with MC, standard TPT, and improved TPT. The agreement between the methods is good at lower temperatures but deteriorates at higher temperatures.

#### 4.4.2. Complete Phase Diagram

**Figure 9:**
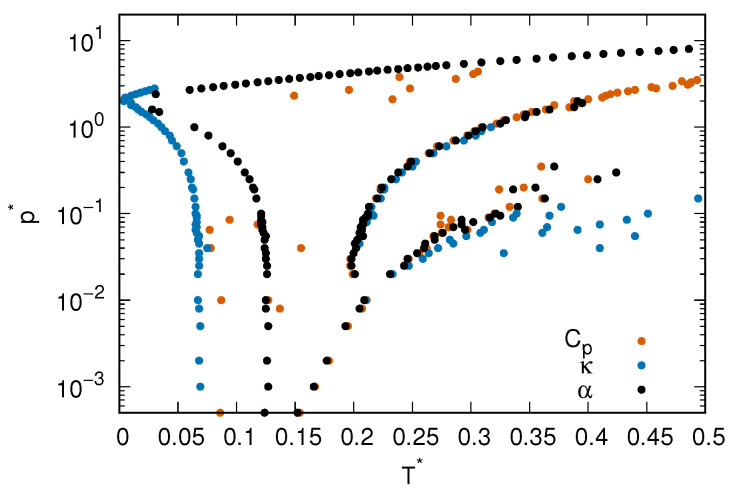
Pressure-temperature phase diagram of the Rose model with real parametrization obtained from extrema of different thermodynamic properties calculated using nested sampling. The points represent extrema in different quantities. 32 particles were used in nested sampling.

We also wanted to determine a complete phase diagram of the Rose model [64]. Nested sampling seemed to be a promising method, as it has been used previously to determine phase transitions in different molecular systems. Nested sampling was used to determine the phase transitions based on the extremes of different thermodynamic functions (heat capacity, isothermal compressibility, thermal expansion coefficient), which usually increase in the presence of a phase transition due to increased fluctuations. The phase diagram of the Rose model with real parameterization, which was determined using nested sampling, is shown in Figure 9. Some of the lines in the diagram are indeed correctly predicted phase transitions (horizontal line at a pressure around 2, the lines at a temperature above 0.15), but some of the lines appear to be artifacts of the method (vertical lines below temperature 0.15). Using nested sampling, we were able to determine some phase transitions, but not all of them, especially not the solid–solid phase transitions. Therefore, we wanted to develop a method that would allow automatic determination of the phase diagram.

In order to develop an automatic approach for the determination of phase diagrams, we first needed a reference diagram with which the different results could be compared. To obtain such a diagram, the angular distribution functions between the molecules were calculated using MD simulations and, based on the differences in the distribution functions, we manually divided the phase space into different phases.

**Figure 10:**
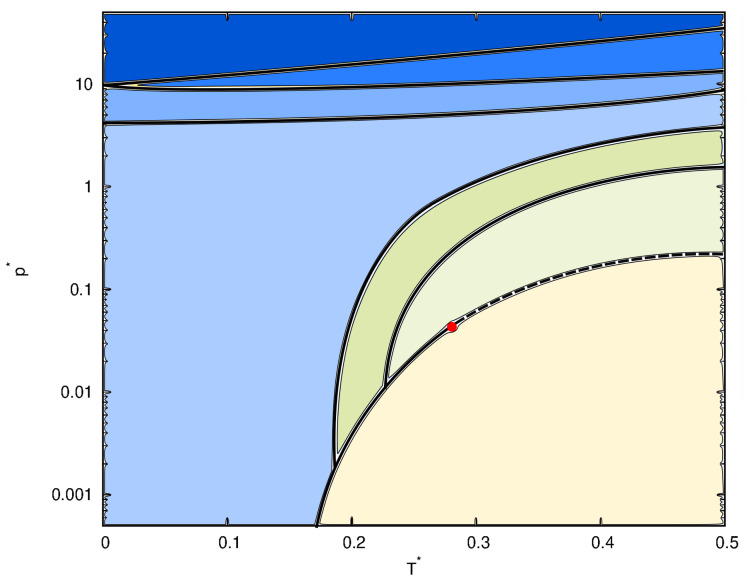
Pressure-temperature phase diagram of the Rose model with real parametrization obtained manually from angular distribution functions. The diffusion data from the simulation helped us to locate the liquid phases. Full lines represnet phase transitions and dash line represents Widom line. The red dot represents critical point.

In Figure 10, a phase diagram of the Rose water model with real parametrization is shown. The blue shades indicate solid phases, the green shades liquid phases, and the yellow color indicates the gaseous phase. The red dot marks the critical point.

Different combinations of unsupervised machine learning methods were used to determine the phase diagram in a more automatic way [64]. The idea was to take a large data set for each phase point and then use dimensionality reduction methods to reduce the dimensions to 3D. Clustering algorithms were then used to group the phase points into phases based on their position in the new 3D space. The dimensionality reduction methods used are Isomap, MDS, t-SNE, and spectral embedding. The clustering algorithms used are k-means, hierarchical clustering, and DBSCAN. Two different datasets were used as input for the unsupervised machine learning. The first dataset was angular distribution functions, and the second dataset was a combination of thermodynamic, dynamic, and structural properties. Both datasets were obtained as a result of standard MD simulations. To determine how successful the different methods were in determining the phase diagram, the machine learning results were compared with the reference diagram.

**Figure 11:**
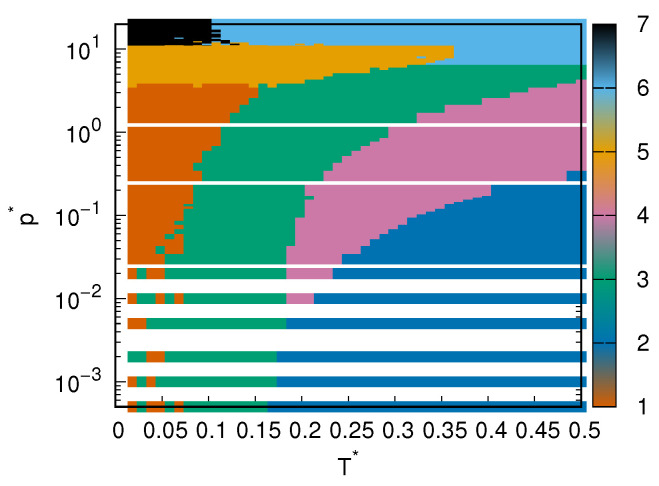
Pressure-temperature phase diagram of the Rose model with real parametrization obtained from angular distribution functions from MD simulations. Isomap was used to decrease the dimensionality of the data, and then the k-means clustering algorithm was used to cluster the data.

When using angular distribution functions as an input dataset, the most successful combination of methods was the isomap for dimensionality reduction and k-means for clustering. The phase diagram obtained with this combination of unsupervised machine learning methods is shown in Figure 11. The liquid and gaseous phases were successfully determined with this combination of methods and agree well with the reference diagram; the transition to solid high-pressure phases is also successfully predicted. On the other hand, the separation of the solid phases is slightly different here compared to the reference diagram. The algorithm separates the main solid phase into two parts. The problem with this is that it is sometimes difficult to recognize whether two separate areas are really two different phases or just one phase with a slightly different structure due to different conditions.

**Figure 12:**
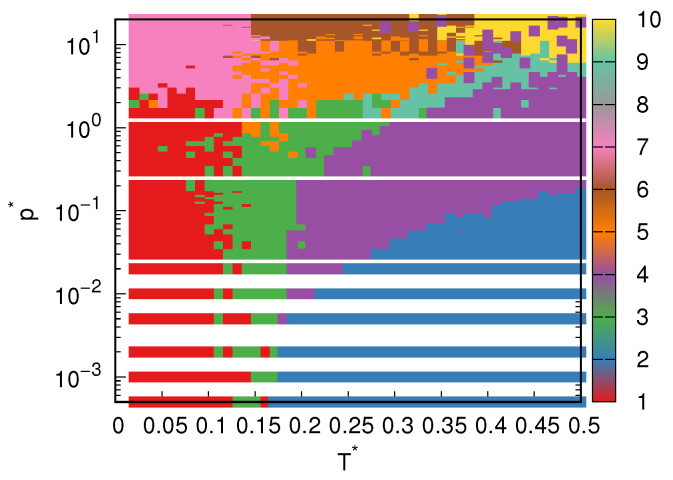
Pressure-temperature phase diagram of Rose model with real parametrization obtained from thermodynamic, structural, and dynamic data from MD simulations. First, DBSCAN and the diffusion coefficient were used to determine solid, liquid, and gas phase, and then T-SNE in combination with the hierarchical clustering algorithm was used to separate solid phases.

The second data set used is a combination of thermodynamic, dynamic, and structural properties calculated with MD. Initially, the same approach was taken as before for the angular distribution functions. This means that all data were used as input for the dimensionality reduction. However, the results were not the best, as the large differences between gaseous, liquid and solid phases overshadowed the differences between different solid phases. To solve this problem, the process was split into two parts. First, the solid, liquid, and gaseous phases were separated with DBSCAN based on the diffusion coefficient. Then the entire dataset (without the diffusion coefficient) was used in combination with machine learning methods. The combinations of machine learning methods were the same as before, but there was no combination that performed exceptionally well. The best quantitative result was achieved with the combination of t-SNE and hierarchical clustering. The phase diagram determined with this combination of methods is shown in Figure 12. Here, the solid, liquid, and gaseous phases are successfully separated; moreover, the solid phases with high density are also separated from the main solid phase, but the position of the transition between them is different from the reference diagram. The problem with the separation of the solid phases is due to the fact that the solid phases of the Rose model with real parametrization are very similar. The similarity of the phases is a consequence of only one characteristic interaction distance and the ability to form hydrogen half-bonds. In all cases, the phases are variations of a hexagonal dense packing, which is more or less compressed depending on the conditions and has more or less empty interstitial sites. The question of whether the Rose model with real parametrization actually has several different solid phases or just one phase that changes depending on the conditions therefore remains unanswered.

### 4.5. Comparison of the Rose Water Model with Real Water and Other 2D Models

The two-dimensional model used in this work is designed to have properties similar to those of real water. Because it is a relatively simple, two-dimensional model, we cannot expect it to quantitatively predict the properties of real water. However, due to its mechanistic design, whose main feature is the formation of hydrogen bonds, this model is still similar enough to real water that we can use it to study various trends in properties that qualitatively match those observed in real water.

**Figure 13:**
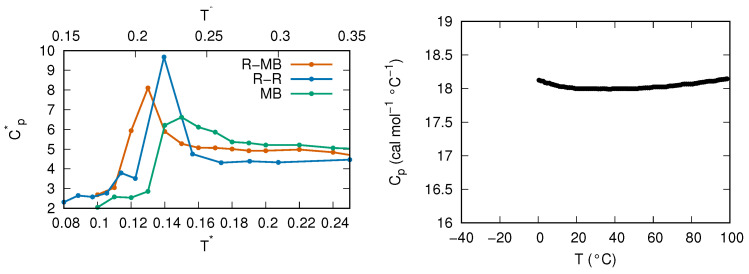
Heat capacity as a function of temperature. The left side shows the values for 2D models (Rose model with MB and real parameterization, and MB model), while the right side shows experimental data [8,53]. In the graphs on the left, the lower x-axis corresponds to the MB model and the Rose model with MB parameterization, while the upper x-axis corresponds to the Rose model with real parameterization.

**Figure 14:**
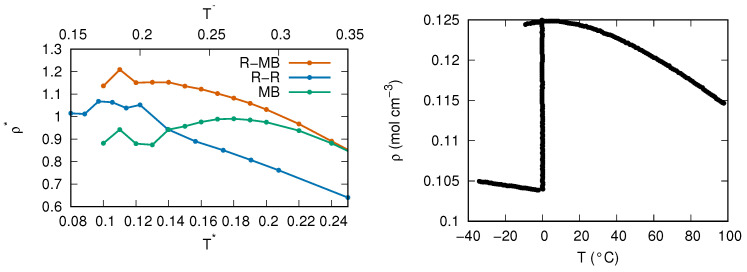
Density as a function of temperature. Different results are labeled as in Figure 13.

**Figure 15:**
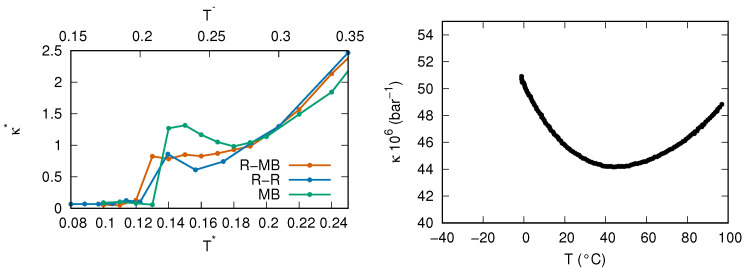
Isothermal compressibility as a function of temperature. Different results are labeled as in Figure 13.

**Figure 16:**
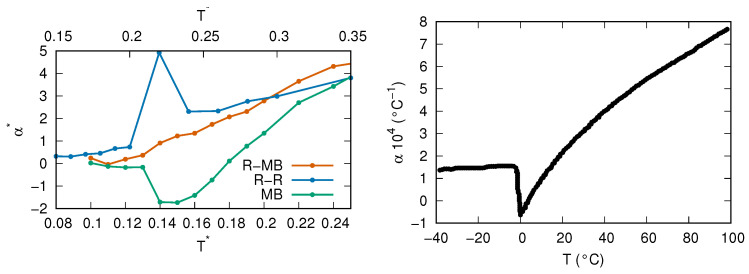
Thermal expansion coefficient as a function of temperature. Different results are labeled as in Figure 13.

Figure 13, Figure 14, Figure 15 and Figure 16 show the temperature dependencies of heat capacity, density, isothermal compressibility, and thermal expansion coefficient for the Rose model with MB and real parameterizations, as well as for the MB model at a pressure of 0.19 (in reduced units). For comparison, the same properties are also shown for real water. The thermodynamic properties of the models do not quantitatively match those of real water, but certain trends are similar. Real water has a high heat capacity in the liquid state, with a minimum at approximately 36 °C. Similarly, 2D models also predict a high heat capacity in the liquid state. The heat capacity of the Rose model has a minimum of 0.2 with MB parameterization and 0.26 with real parameterization. The Rose and MB models qualitatively predict the trend in the density of liquid water, as both models exhibit a density maximum similar to real water. The MB model also successfully predicts a lower density in the solid state compared to the liquid state. Like real water, both parameterizations of the Rose model and the MB model display a minimum in isothermal compressibility as a function of temperature. The thermal expansion coefficient of real water has a minimum at approximately 4 °C, where it also becomes negative. The MB model’s thermal expansion coefficient follows the same trend; however, the Rose model does not predict these anomalous properties as accurately as the MB model. We have shown that even simple 2D models can successfully predict some trends and characteristics of the thermodynamic quantities of real water. Of course, the models differ somewhat in how closely they resemble the behavior of real water.

**Figure 17:**
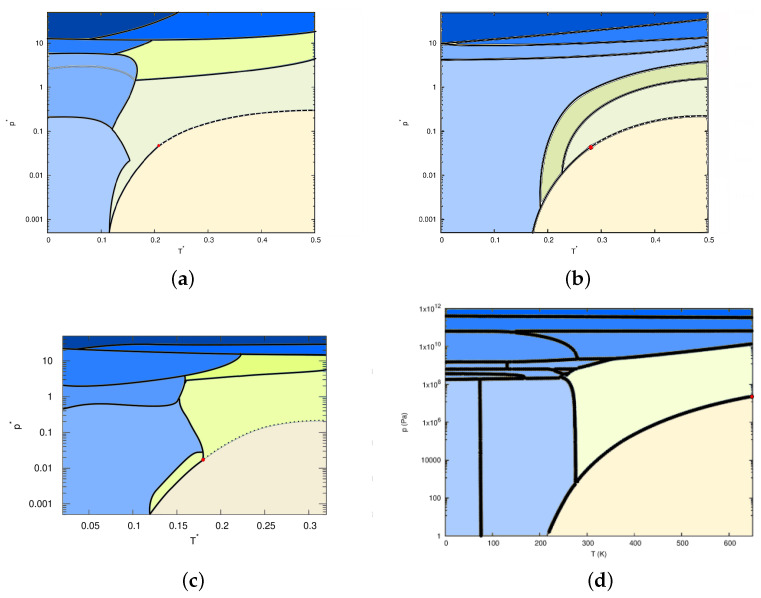
Qualitative comparison of phase diagrams for the Rose model with MB (**a**) and real (**b**) parameterization, the MB model (**c**), and real water [105] (**d**). Critical points are marked with red dots. Blue indicates solid phases, green indicates liquid phases, and orange indicates gaseous phases. Solid lines represnet phase transitions and dash line represents Widom line.

Real water has a complex phase diagram with many different solid phases. Figure 17 shows the phase diagrams of the Rose model with MB and real parameterization, the MB model, and real water. Generally, all the phase diagrams are similar: they have a gaseous phase in the lower right corner (at low pressures and high temperatures), a liquid phase above the gaseous phase, and a solid phase surrounding the liquid phase from the left and above. A quantitative comparison of the positions of the different phases is not meaningful, as the models and real water differ in dimensionality, so the units of measurement (e.g., pressure) are not the same. However, the diagrams can be compared qualitatively in terms of how the different phases are arranged relative to each other. Although some properties of the Rose model with real parameterization are more similar to real water, its phase diagram is the least similar to that of real water among those shown. It has the least variety of solid phases, and nowhere does it have an equilibrium curve between the solid and liquid phases with a negative slope, which is a characteristic of real water. The phase diagrams of the Rose model with MB parameterization and the MB model are more similar to that of real water, as they have a relatively large number of different solid phases, and the distribution of these phases at least resembles the distribution in real water.

In addition, both models have an equilibrium curve between the liquid and solid phases with a negative slope. In the Rose model, part of this equilibrium curve lies below the critical point, which is consistent with real water, whereas the MB model does not have an equilibrium curve with a negative slope below the critical point. The dimensionality of the system significantly impacts the structure, as phases with a characteristic three-dimensional distribution cannot form in two dimensions. As a result, two-dimensional models cannot directly describe the phase behavior of real water, since the phase structures differ. However, a two-dimensional model can help interpret the phase behavior of real water, as a two-dimensional system is easier to visualize and allows direct observation of structural changes. For example, a two-dimensional model can be used to observe how different conditions affect the distribution of interstitial sites in the structure. In a 2D system, it is easier to observe trends in structural changes and quantify them in a model, which can then be adapted for real water. The model would still need adjustment, but the basic mechanism of behavior would likely be similar.

## 5. Conclusions

In this work, we have reviewed research on the Rose water model. The Rose water model has two widely used parameterizations: the MB parameterization, which is similar to the Mercedes–Benz water model, and the real parameterization, which more closely resembles experimental water. Here, we focused on the real parameterization of the model. The Rose water model provides insight into the molecular mechanisms underlying various thermodynamic, dynamic, and structural properties of water. Many of water’s anomalous properties arise from its ability to form hydrogen bonds. Using the Rose water model, the strong orientational dependence of the hydrogen bond is successfully captured, allowing the model to replicate key anomalous properties of real water. Monte Carlo and molecular dynamics simulations were performed to calculate different properties of the Rose water model under various conditions. The simulation data served as a reference point for comparison with results from more analytical theories. Overall, the theories reproduce the properties of the Rose water model as predicted by simulation, with the agreement between methods being sometimes quantitative and sometimes qualitative, depending on the property and conditions. The Rose water model has also been successfully used as a testbed for developing new computational approaches, such as a more efficient and automated method for determining phase diagrams and a semi-analytical structural model of water.

## Data Availability

Data is available per request.

## References

[1] Franks F. (2000). Water: A Matrix of Life.

[2] Prielmeier F.X., Lang E.W., Speedy R.J., Lüdemann H.D. (1987). Diffusion in supercooled water to 300 MPa. Phys. Rev. Lett..

[3] Prielmeier F.X., Lang E.W., Speedy R.J., Lüdemann H.D. (1988). The Pressure Dependence of Self Diffusion in Supercooled Light and Heavy Water. Berichte Bunsenges. Phys. Chem..

[4] Angell C.A., Finch E.D., Bach P. (1976). Spin-echo diffusion coefficients of water to 2380 bar and −20 °C. J. Chem. Phys..

[5] de Oliveira A.B., Franzese G., Netz P.A., Barbosa M.C. (2008). Waterlike hierarchy of anomalies in a continuous spherical shouldered potential. J. Chem. Phys..

[6] de Oliveira A.B., Netz P.A., Barbosa M.C. (2009). An ubiquitous mechanism for water-like anomalies. EPL Europhys. Lett..

[7] Fomin Y.D., Tsiok E.N., Ryzhov V.N. (2011). Inversion of sequence of diffusion and density anomalies in core-softened systems. J. Chem. Phys..

[8] Eisenberg D., Kauzmann W. (1969). The Structure and Properties of Water.

[9] Marechal Y. (2007). The Hydrogen Bond and the Water Molecule.

[10] Salzmann C.G. (2019). Advances in the experimental exploration of water’s phase diagram. J. Chem. Phys..

[11] Agarwal M., Alam M.P., Chakravarty C. (2011). Thermodynamic, Diffusional, and Structural Anomalies in Rigid-Body Water Models. J. Phys. Chem. B.

[12] Stanley H.E., Buldyrev S.V., Giovambattista N., Nave E.L., Mossa S., Scala A., Sciortino F., Starr F.W., Yamada M. (2003). Application of Statistical Physics to Understand Static and Dynamic Anomalies in Liquid Water. J. Stat. Phys..

[13] Russo J., Akahane K., Tanaka H. (2018). Water-like anomalies as a function of tetrahedrality. Proc. Natl. Acad. Sci. USA.

[14] Jabes B.S., Agarwal M., Chakravarty C. (2010). Tetrahedral order, pair correlation entropy, and waterlike liquid state anomalies: Comparison of GeO_2_ with BeF_2_, SiO_2_, and H_2_O. J. Chem. Phys..

[15] Angell C.A., Bressel R.D., Hemmati M., Sare E.J., Tucker J.C. (2000). Water and its anomalies in perspective: Tetrahedral liquids with and without liquid-liquid phase transitions. Phys. Chem. Chem. Phys..

[16] Hoang V.V., Anh N.H.T., Zung H. (2007). Liquid–liquid phase transition and anomalous diffusion in simulated liquid GeO_2_. Phys. B Condens. Matter.

[17] Agarwal M., Sharma R., Chakravarty C. (2007). Ionic melts with waterlike anomalies: Thermodynamic properties of liquid BeF_2_. J. Chem. Phys..

[18] Agarwal M., Chakravarty C. (2007). Waterlike Structural and Excess Entropy Anomalies in Liquid Beryllium Fluoride. J. Phys. Chem. B.

[19] Morishita T. (2005). Anomalous diffusivity in supercooled liquid silicon under pressure. Phys. Rev. E.

[20] Poole P.H., Hemmati M., Angell C.A. (1997). Comparison of Thermodynamic Properties of Simulated Liquid Silica and Water. Phys. Rev. Lett..

[21] Sharma R., Chakraborty S.N., Chakravarty C. (2006). Entropy, diffusivity, and structural order in liquids with waterlike anomalies. J. Chem. Phys..

[22] Fumagalli L., Esfandiar A., Fabregas R., Hu S., Ares P., Janardanan A., Yang Q., Radha B., Taniguchi T., Watanabe K. (2018). Anomalously low dielectric constant of confined water. Science.

[23] Algara-Siller G., Lehtinen O., Wang F.C., Nair R.R., Kaiser U., Wu H.A., Geim A.K., Grigorieva I.V. (2015). Square ice in graphene nanocapillaries. Nature.

[24] Kapil V., Schran C., Zen A., Chen J., Pickard C.J., Michaelides A. (2022). The first-principles phase diagram of monolayer nanoconfined water. Nature.

[25] Rżysko W., Patrykiejew A., Sokołowski S., Pizio O. (2010). Phase behavior of a two-dimensional and confined in slitlike pores square-shoulder, square-well fluid. J. Chem. Phys..

[26] Koga K., Tanaka H. (2005). Phase diagram of water between hydrophobic surfaces. J. Chem. Phys..

[27] Zhao W.H., Wang L., Bai J., Yuan L.F., Yang J., Zeng X.C. (2014). Highly Confined Water: Two-Dimensional Ice, Amorphous Ice, and Clathrate Hydrates. Acc. Chem. Res..

[28] Gao Z., Giovambattista N., Sahin O. (2018). Phase Diagram of Water Confined by Graphene. Sci. Rep..

[29] Kaneko T., Bai J., Yasuoka K., Mitsutake A., Zeng X.C. (2014). Liquid-solid and solid-solid phase transition of monolayer water: High-density rhombic monolayer ice. J. Chem. Phys..

[30] Zangi R., Mark A.E. (2003). Monolayer Ice. Phys. Rev. Lett..

[31] Verhagen T., Klimes J., Pacakova B., Kalbac M., Vejpravova J. (2020). Anomalous Freezing of Low-Dimensional Water Confined in Graphene Nanowrinkles. ACS Nano.

[32] Ghorbanfekr H., Behler J., Peeters F.M. (2020). Insights into Water Permeation through hBN Nanocapillaries by Ab Initio Machine Learning Molecular Dynamics Simulations. J. Phys. Chem. Lett..

[33] Kumar P., Buldyrev S.V., Starr F.W., Giovambattista N., Stanley H.E. (2005). Thermodynamics, structure, and dynamics of water confined between hydrophobic plates. Phys. Rev. E.

[34] Corsetti F., Matthews P., Artacho E. (2016). Structural and configurational properties of nanoconfined monolayer ice from first principles. Sci. Rep..

[35] Bianco V., Franzese G. (2014). Critical behavior of a water monolayer under hydrophobic confinement. Sci. Rep..

[36] Bernal J.D., Fowler R.H. (1933). A theory of water and ionic solution, with particular reference to hydrogen and hydroxyl ions. J. Chem. Phys..

[37] Barker J.A., Watts R.O. (1969). Structure of water; A Monte Carlo calculation. Chem. Phys. Lett..

[38] Jorgensen W.L. (1981). Transferable Intermolecular Potential Functions for Water, Alcohols, and Ethers. Application to Liquid Water. J. Am. Chem. Soc..

[39] Jorgensen W.L., Chandrasekhar J., Madura J.D., Impey R.W., Klein M.L. (1983). Comparison of simple potential functions for simulating liquid water. J. Chem. Phys..

[40] Mahoney M.W., Jorgensen W.L. (2000). A five-site model for liquid water and the reproduction of the density anomaly by rigid, nonpolarizable potential functions. J. Chem. Phys..

[41] Jorgensen W.L. (1982). Revised TIPS for simulations of liquid water and aqueous solutions. J. Chem. Phys..

[42] Abascal J.L.F., Vega C. (2005). A general purpose model for the condensed phases of water: TIP4P/2005. J. Chem. Phys..

[43] Horn H.W., Swope W.C., Pitera J.W. (2005). Characterization of the TIP4P-Ew water model: Vapor pressure and boiling point. J. Chem. Phys..

[44] Berendsen H.J.C., Postma J.P.M., van Gunsteren W.F., Hermans J. (1981). Interaction Models for Water in Relation to Protein Hydration.

[45] Berendsen H.J., Grigera J.R., Straatsma T.P. (1987). The missing term in effective pair potentials. J. Phys. Chem..

[46] Cheng B., Engel E.A., Behler J., Dellago C., Ceriotti M. (2019). Ab initio thermodynamics of liquid and solid water. Proc. Natl. Acad. Sci. USA.

[47] Chen M., Ko H.Y., Remsing R.C., Andrade M.F.C., Santra B., Sun Z., Selloni A., Car R., Klein M.L., Perdew J.P. (2017). Ab initio theory and modeling of water. Proc. Natl. Acad. Sci. USA.

[48] Eastman P., Galvelis R., Peláez R.P., Abreu C.R.A., Farr S.E., Gallicchio E., Gorenko A., Henry M.M., Hu F., Huang J. (2024). OpenMM 8: Molecular Dynamics Simulation with Machine Learning Potentials. J. Phys. Chem. B.

[49] Dell’Angelo D., Lainé J., Said H., Foucaud Y., Badawi M. (2024). Machine Learning Force Field beyond the Limits of Classical and First-Principles Molecular Dynamics Simulations: The Case of Kaolinite Hydration. J. Phys. Chem. C.

[50] Omranpour A., Hijes P.M.D., Behler J., Dellago C. (2024). Perspective: Atomistic simulations of water and aqueous systems with machine learning potentials. J. Chem. Phys..

[51] Gao R., Li Y., Car R. (2024). Enhanced deep potential model for fast and accurate molecular dynamics: Application to the hydrated electron. Phys. Chem. Chem. Phys..

[52] Ben-Naim A. (1971). Statistical mechanics of “waterlike” particles in two dimensions. I. Physical model and application of the Percus-Yevick equation. J. Chem. Phys..

[53] Silverstein K.A., Haymet A.D., Dill K.A. (1998). A simple model of water and the hydrophobic effect. J. Am. Chem. Soc..

[54] Williamson C.H., Hall J.R., Fennell C.J. (2017). Two-dimensional molecular simulations using rose potentials. J. Mol. Liq..

[55] Molinero V., Moore E.B. (2009). Water Modeled As an Intermediate Element between Carbon and Silicon. J. Phys. Chem. B.

[56] Coronas L.E., Franzese G. (2024). Phase behavior of metastable water from large-scale simulations of a quantitatively accurate model near ambient conditions: The liquid–liquid critical point. J. Chem. Phys..

[57] Ogrin P., Urbic T., Fennell C.J. (2022). Statistical-mechanical liquid theories reproduce anomalous thermodynamic properties of explicit two-dimensional water models. Phys. Rev. E.

[58] Ogrin P., Urbic T. (2023). Angle-dependent integral equation theory improves results of thermodynamics and structure of rose water model. J. Chem. Phys..

[59] Ogrin P., Urbic T. (2024). Effect of nonpolar solute on the local structure of aqueous solution: Orientation-dependent integral equation study of nonpolar solute in rose water model. J. Mol. Liq..

[60] Ogrin P., Urbic T. (2023). Thermodynamics perturbation theory for solvation of nonpolar solutes in rose model. Phys. Rev. E.

[61] Ogrin P., Urbic T. (2023). Hierarchy of anomalies in the simple rose model of water. J. Mol. Liq..

[62] Ogrin P., Urbic T. (2022). Liquid-vapour coexistence line and percolation line of rose water model. J. Mol. Liq..

[63] Ogrin P., Urbic T. (2024). The phase diagram of Mercedes Benz model of water using nested sampling algorithm and molecular dynamics simulations. Fluid Phase Equilibria.

[64] Ogrin P., Urbic T. (2025). Calculating a Phase Diagram of a Simple Water Model Using Unsupervised Machine Learning on Simulation Data. J. Chem. Theory Comput..

[65] Ogrin P., Urbic T. (2022). Rose water in random porous media: Associative replica Ornstein-Zernike theory study. J. Mol. Liq..

[66] Ogrin P., Urbic T. (2023). Simple rose model of water in constant electric field. Phys. Rev. E.

[67] Ogrin P., Urbic T. (2025). From hydrogen bonding to resonance: A molecular dynamics study of the rose water model in an alternating electric field. Phys. Rev. E.

[68] Ogrin P., Urbic T. (2025). Snowflake Model of Water: A Fast Approach for Calculation of Structural Properties of Liquid Water. J. Chem. Theory Comput..

[69] Raveché H.J. (1971). Entropy and Molecular Correlation Functions in Open Systems. I. Derivation. J. Chem. Phys..

[70] Baranyai A., Evans D.J. (1989). Direct entropy calculation from computer simulation of liquids. Phys. Rev. A.

[71] Mittal J., Errington J.R., Truskett T.M. (2006). Relationship between thermodynamics and dynamics of supercooled liquids. J. Chem. Phys..

[72] Mittal J., Errington J.R., Truskett T.M. (2006). Quantitative Link between Single-Particle Dynamics and Static Structure of Supercooled Liquids. J. Phys. Chem. B.

[73] Chakraborty S.N., Chakravarty C. (2007). Entropy, local order, and the freezing transition in Morse liquids. Phys. Rev. E.

[74] Scala A., Starr F.W., Nave E.L., Sciortino F., Stanley H.E. (2000). Configurational entropy and diffusivity of supercooled water. Nature.

[75] Ogrin P., Dias C.L., Urbic T. (2024). Code for molecular dynamics simulation of two dimensional Mercedes-Benz water model. Comput. Phys. Commun..

[76] Swope W.C., Andersen H.C., Berens P.H., Wilson K.R. (1982). A computer simulation method for the calculation of equilibrium constants for the formation of physical clusters of molecules: Application to small water clusters. J. Chem. Phys..

[77] Bussi G., Donadio D., Parrinello M. (2007). Canonical sampling through velocity rescaling. J. Chem. Phys..

[78] Berendsen H.J.C., Postma J.P.M., van Gunsteren W.F., DiNola A., Haak J.R. (1984). Molecular dynamics with coupling to an external bath. J. Chem. Phys..

[79] Bernetti M., Bussi G. (2020). Pressure control using stochastic cell rescaling. J. Chem. Phys..

[80] Wertheim M.S. (1986). Fluids with highly directional attractive forces. III. Multiple attraction sites. J. Stat. Phys..

[81] Wertheim M.S. (1986). Fluids with highly directional attractive forces. IV. Equilibrium polymerization. J. Stat. Phys..

[82] Wertheim M.S. (1987). Thermodynamic perturbation theory of polymerization. J. Chem. Phys..

[83] Urbič T., Vlachy V., Kalyuzhnyi Y.V., Southall N.T., Dill K.A. (2000). A two-dimensional model of water: Theory and computer simulations. J. Chem. Phys..

[84] Scalise O.H., Zarragoicoechea G.J., Gonzalez L.E., Silbert M. (1998). Phase equilibria of the two-dimensional Lennard-Jones fluid: Reference systems and perturbation theories. Mol. Phys..

[85] Hansen J.P., McDonald I. (2006). Theory of Simple Liquids.

[86] Urbič T., Vlachy V., Kalyuzhnyi Y.V., Dill K.A. (2007). An improved thermodynamic perturbation theory for Mercedes-Benz water. J. Chem. Phys..

[87] Wertheim M.S. (1984). Fluids with highly directional attractive forces. I. Statistical thermodynamics. J. Stat. Phys..

[88] Wertheim M.S. (1984). Fluids with highly directional attractive forces. II. Thermodynamic perturbation theory and integral equations. J. Stat. Phys..

[89] Wertheim M.S. (1986). Fluids of dimerizing hard spheres, and fluid mixtures of hard spheres and dispheres. J. Chem. Phys..

[90] Urbič T., Vlachy V., Kalyuzhnyi Y.V., Southall N.T., Dill K.A. (2002). A two-dimensional model of water: Solvation of nonpolar solutes. J. Chem. Phys..

[91] Barker J.A., Henderson D. (1967). Perturbation theory and equation of state for fluids. II. A successful theory of liquids. J. Chem. Phys..

[92] Kalyuzhnyi Y.V., Cummings P.T. (1996). On the relation between the Wertheim’s two-density integral equation theory for associating fluids and Chandler-Silbey-Ladanyi integral equation theory for site-site molecular fluids. J. Chem. Phys..

[93] Nezbeda I., Smith W.R., Kolafa J. (1994). Molecular theory of phase equilibria in model associated mixtures. I. Binary mixtures of water and a simple fluid. J. Chem. Phys..

[94] Chang J., Sandler S.I. (1995). The correlation functions of hard-sphere chain fluids: Comparison of the Wertheim integral equation theory with the Monte Carlo simulation. J. Chem. Phys..

[95] Vakarin E.V., Duda Y.J., Holovko M.F. (1997). Integral equation theory for the four bonding sites model of I. Structure factor and compressibility associating fluids. Mol. Phys..

[96] Talman J.D. (1978). Numerical Fourier and Bessel transforms in logarithmic variables. J. Comput. Phys..

[97] Urbič T., Vlachy V., Kalyuzhnyi Y.V., Dill K.A. (2003). Orientation-dependent integral equation theory for a two-dimensional model of water. J. Chem. Phys..

[98] Ward D., Lado F. (1988). Structure, thermodynamics, and orientational correlations of the nematogenic hard ellipse fluid from the Percus-Yevick equation. Mol. Phys..

[99] Ward D., Lado F. (1988). Percus-Yevick solutions for the planar dumbell fluid. Mol. Phys..

[100] Rick S.W., Haymet A.D.J. (1989). Density functional theory for the freezing of Lennard-Jones binary mixtures. J. Chem. Phys..

[101] Urbič T., Vlachy V., Kalyuzhnyi Y.V., Dill K.A. (2007). Theory for the solvation of nonpolar solutes in water. J. Chem. Phys..

[102] Urbic T., Dill K.A. (2010). A statistical mechanical theory for a two-dimensional model of water. J. Chem. Phys..

[103] Truskett T.M., Dill K.A. (2002). A Simple Statistical Mechanical Model of Water. J. Phys. Chem. B.

[104] Urbic T., Dill K.A. (2023). Simple Model of Liquid Water Dynamics. J. Phys. Chem. B.

[105] Chaplin M.F. (2019). Structure and Properties of Water in Its Various States.

